# Evolution of mammalian migrations for refuge, breeding, and food

**DOI:** 10.1002/ece3.3120

**Published:** 2017-06-22

**Authors:** Gitanjali E. Gnanadesikan, William D. Pearse, Allison K. Shaw

**Affiliations:** ^1^ Department of Ecology and Evolutionary Biology Princeton University Princeton NJ USA; ^2^ Department of Ecology, Evolution, and Behavior University of Minnesota St. Paul MN USA; ^3^ Department of Biology McGill University Montréal QC Canada; ^4^ Département des Sciences Biologiques Université du Québec à Montréal Montréal QC Canada; ^5^ Division of Evolution, Ecology and Genetics, Research School of Biology Australian National University Canberra ACT Australia; ^6^ Department of Biology & Ecology Center Utah State University Logan UT USA; ^7^ Present address: School of Anthropology University of Arizona Tucson AZ USA

**Keywords:** body mass, conservation, diet, IUCN Red List, movement ecology, seasonal migration, tracking

## Abstract

Many organisms migrate between distinct habitats, exploiting variable resources while profoundly affecting ecosystem services, disease spread, and human welfare. However, the very characteristics that make migration captivating and significant also make it difficult to study, and we lack a comprehensive understanding of which species migrate and why. Here we show that, among mammals, migration is concentrated within Cetacea and Artiodactyla but also diffusely spread throughout the class (found in 12 of 27 orders). We synthesize the many ecological drivers of round‐trip migration into three types of movement—between breeding and foraging sites, between breeding and refuge sites, and continuous tracking of forage/prey—each associated with different traits (body mass, diet, locomotion, and conservation status). Our results provide only partial support for the hypothesis that migration occurs without phylogenetic constraint. Furthermore, our findings suggest that categorizing migration into these three types may aid predictions of migrants’ responses to environmental changes.

## 1 INTRODUCTION

The migratory movement of organisms across the globe is one of the most charismatic and visually alluring biological phenomena. Although there is no universally agreed upon definition of migration (Dingle, 2014), here we consider migration as the predictable, round‐trip, seasonal movement of organisms between two or more locations. Migrants provide vital links between habitats that are physically or ecologically distant, transporting nutrients, propagules, and pathogens (Bauer & Hoye, 2014; Webster, Marra, Haig, Bensch, & Holmes, 2002). Humans are influenced by many migratory species, including crop pests, commercially fished species, birds transmitting avian influenza, and ungulates that compete with domestic livestock (Altizer, Bartel, & Han, 2011; Dingle, 2014). Anthropogenic climatic and environmental changes are especially likely to affect migratory species (Robinson et al., 2009), and there is increasing concern over whether and how migrants will respond (Dingle, 2014; Lindström et al., 2014), especially since migration loss can have secondary implications at both the species level (population decline and increased infection risk; Bolger, Newmark, Morrison, & Doak, 2008; Satterfield, Maerz, & Altizer, 2015) and ecosystem level (reduced nutrient inputs; Gresh, Lichatowich, & Schoonmaker, 2000). Despite the importance of understanding migration, the large spatiotemporal scales that migration encompasses, by definition, make it difficult to study (Webster et al., 2002), and most migration studies focus on one species, habitat, or other aspect of migration. The ability to integrate—within a species, across clades, and spanning methodological approaches—is currently one of the grand challenges of migration biology (Bolger et al., 2008; Bowlin et al., 2010).

We still lack a unified understanding of which species migrate and why they do so. Although it has been suggested that migration occurs without phylogenetic constraint (Alerstam, Hedenström, & Akesson, 2003), particularly across birds (Salewski & Bruderer, 2007), it is unclear to what extent this is true for other taxonomic groups, and studies of migration are often limited to groups known to be highly migratory. The many potential benefits associated with migration include thermoregulation, increasing energetic gain, avoiding inhospitable climates, minimizing competition, seeking mates and breeding sites, and avoiding parasitism, pathogens, and predation (Avgar, Street, & Fryxell, 2014; Chapman, Reynolds, & Wilson, 2015; Northcote, 1978; Russell, Bauer, Johnson, & Elewa, 2005; Wolcott & Wolcott, 1985). We synthesize these factors into a framework of three types of round‐trip migration, which we term “breeding,” “refuge,” and “tracking” migrations (Table 1, Shaw, 2016). Breeding migrants reproduce in one location and forage in another, migrating each time they reproduce (e.g., humpback whales move between high‐latitude feeding grounds and low‐latitude breeding grounds; Craig & Herman, 1997). Refuge migrants breed in the same place they primarily forage, but migrate away to escape seasonally unfavorable conditions (e.g., Florida manatees migrate north as temperatures warm and return south as the water cools; Reynolds & Odell, 1991). Tracking migrants move relatively continuously, following required nutritional resources (e.g., wildebeest track vegetation gradients in a loop; Boone, Thirgood, & Hopcraft, 2006).

**Table 1:** The ecological drivers of migration, distilled into three types (all photos are Public Domain)

Type	Specific benefits	Example	
Breeding	Mating Avoid predation of young	Caspian seal (*Pusa caspica*): Move north in late autumn to breed on the ice, while foraging throughout the sea during the rest of the year (Härkönen, 2008).	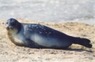
Refuge	Thermoregulation Suitable hibernation sites Avoid parasitism or predation Avoid flooding, snow, or fires	Gray myotis (*Myotis grisescens*): Fly over 100 km to one of nine caves suitable for hibernation (temperature and humidity being major factors) (Wilson & Ruff, 1999).	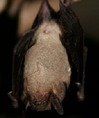
Tracking	Increase quantity of nutrients, forage, and prey Increase quality of nutrients, forage, and prey Avoid nutrient depletion by conspecifics Increase water availability	Cheetah (*Acinonyx jubatus*): Follow migratory Thomson's gazelles (partial migration) (Durant et al., 1988).	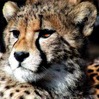

Here, we test the hypothesis that migration (including partial migration) is not phylogenetically constrained across mammals. The over 5,400 extant mammals are a tractable clade for studying migration as they are historically well‐studied despite relatively few, highly visible species (e.g., wildebeest) having been examined with respect to migration *per se* (Harris, Thirgood, Hopcraft, Cromsigt, & Berger, 2009). The diversity of mammalian locomotion types (walking, swimming, flying), habitats (terrestrial, freshwater, marine; Schipper et al., 2008), and diets (carnivore, omnivore, herbivore) permits examination of the importance of biophysical constraints on the evolution of migration. Evidence suggests that mammal migrations are declining (Bolger et al., 2008). To develop a comprehensive understanding of what factors threaten these migrations, we must first understand why mammals migrate (Harris et al., 2009). Although previous studies have summarized migration for subsets of mammals (e.g., Avgar et al., 2014; Bisson, Safi, & Holland, 2009; Harris et al., 2009), none has synthesized migratory patterns for all mammals, as we do here. This systematic approach avoids assumptions regarding which species or clades are migratory and permits the first comprehensive analysis of phylogenetic patterns of mammalian migration.

## 2 MATERIALS AND METHODS

The migration data we collected as well as the scripts used to conduct our analyses are available from the Dryad Digital Repository: http://dx.doi.org/10.5061/dryad.78v5j (Gnanadesikan, Pearse, & Shaw, 2017).

### 2.1 Data collection

We used the IUCN Red List as our comprehensive set of mammal species (5,420 extant species; http://iucnredlist.org; exported on 23 January 2013). For each species, we searched for relevant information on either movement patterns or lack thereof, drawing from primary literature through Google Scholar and Web of Science searches (with binomial names taken from the IUCN list, and keywords “migration,” “movement,” “home range,” “sedentary,” “season”), articles published in *Mammalian Species* (http://mspecies.oxfordjournals.org), and Appendices I and II of the Convention on Migratory Species (www.cms.int/pdf/en/CMS_Species_6lng.pdf, downloaded February 2012). When primary sources were not available, we used information from IUCN fact sheets, the PanTHERIA database (Jones et al., 2009), the Animal Diversity Web database (Myers et al., 2015), and regional guidebooks. Overall, we incorporated information from over 3,500 sources.

### 2.2 Classifying movement patterns

Next, we classified each species into one of four categories: (1) Migratory: Species for which there was a documented, clear, predictable, and regular movement on a seasonal (or longer) time scale, for at least some individuals (this would include partially migratory species); (2) nonmigratory: Species for which there was no evidence of seasonal movement, and there was documentation of individuals occupying the same location year‐round (e.g., data on an annual home range); (3) possibly migratory: Species for which there was some evidence of seasonal movement or regular fluctuations in local population size, which could be indicative of migration but could also be attributed to a nonmigratory behavior (e.g., seasonally variable breeding, irruptive events); and (4) data deficient: Species that did not have sufficient details to be classified into one of these above categories. If there was documentation of both migratory and nonmigratory behavior for a species (e.g., partial migration where only a fraction of the population migrates), we classified it as migratory. If there was any documentation of a historical migration in a species that had since been disrupted (e.g., by human activities), we classified the species as migratory. If there were only anecdotal reports, we classified the species as possibly migratory. Only definitively migratory and nonmigratory species (Fig. S1a in Appendix S1) were included in the subsequent phylogenetic and regression analyses.

### 2.3 Defining migration

For the purpose of this study, we define migration as a round‐trip movement, on a seasonal or annual schedule, that is significantly longer distance than daily movements and allows an individual to make use of different resources in different locations. Movements classified as migration under this definition (although a subset of all possible “migrations” as considered by some; Dingle & Drake, 2007) share certain commonalities, particularly on temporal and spatial scales, that make a synthesis and pattern analysis appropriate. Unidirectional, daily, multigenerational, and local movements were all considered not comparable. We considered movements to be “significantly longer” if they were at least an order of magnitude longer than normal activity movements during the rest of the year. We used this relative measure of distance (rather than absolute) to allow for the inclusion of cases where individuals move along ecological gradients that occur over short scales—for example, migrations that occur along altitudinal gradients or between water and land. There is still, however, considerable uncertainty associated with average versus maximum recorded migrations and whether these maximum records constitute mere outliers or actual differences in behavior among populations. To this end, the category of “possibly migratory” was used to designate cases where there was evidence of seasonal movements but not enough information to determine whether this behavior constituted migration.

### 2.4 Migration type

Next, we classified each migratory species into three types: breeding, refuge, and tracking (Shaw, 2016). Breeding migrants were those who moved between a breeding site and a feeding site. Breeding sites posed some advantage for mating, rearing, or the survival of the young, rather than meeting other requirements of an adult individual (in extreme cases, adults do not graze or hunt on the breeding grounds). In contrast, feeding sites were those where adults did the majority of their foraging or hunting. In some species, individuals skip migration altogether in years when they do not breed. Refuge sites allow individuals to temporarily escape unsuitable conditions (e.g., predation, temperature, or flooding). Tracking migrants moved between distinct feeding locations (e.g., to follow rainfall‐driven forage, seasonal fruit, or prey that were themselves migratory). Tracking migrations were more continuous than the others, often lacking defined sites, but as per our definition of migration, movements were directed, predictable, and long distance, not simply moving to a new spot when one was overexploited (i.e., we excluded nomadic movements). We were able to classify 180 species (of 235 migrants; Fig S1a‐b in Appendix S1) as exhibiting at least one migration type. Of these, 25 species had migrations that could be classified as two types. In the analyses, these species are added to both categories (e.g., a bat that hibernates in a cave and uses a distinct breeding location would be considered both a breeding and a refuge migrant). We were unable to classify the other 55 migrants, as we could not find information in the literature describing what activities were undertaken by these migrants in each location.

### 2.5 Locomotion

A primary means of long‐distance locomotion (walking, swimming, or flying) was designated for each species. In most cases, this was a straightforward determination given body type and habitat. For migratory species, the type of locomotion used for their migration was used. Semi‐aquatic nonmigratory species were classified as walkers unless there was evidence that a species made long‐distance movements by water (in which case they were classified as swimmers).

### 2.6 Motivation and overview of comparative analyses

The importance of accounting for phylogenetic nonindependence when conducting comparative analyses of traits across species is well established (Cooper, Jetz, & Freckleton, 2010; Freckleton, 2009; Freckleton, Harvey, & Pagel, 2002). Similarly, there is a growing understanding that comparative phylogenetic methods should account for uncertainty in phylogenetic topology and branch lengths by conducting their analyses across a set of candidate phylogenies (Bollback, 2005; Huelsenbeck, Ronquist, Nielsen, & Bollback, 2001). The approach we take therefore is to run all our models across 1000 candidate mammal phylogenies taken from a recent combined molecular and fossil phylogeny for all mammals (Faurby & Svenning, 2015). We then report the mean and standard deviation of all model coefficients from these models. Although there is uncertainty in the placement of many species in these phylogenies, we re‐emphasize that, by conducting our analyses across many phylogenies, we average out this uncertainty (following the advice for comparative analysis in Bollback (2005)). Throughout, all software in *courier italics* refer to R packages and functions (R Core Team 2014).

Our overall approach is to examine the distribution of migrants throughout the mammal tree of life (1), to compare the distributions of different locomotion types (walking, swimming, and flying) and migration (2), and finally to statistically model migration and each of the three kinds of migration as function of life‐history variables (3). We expect that each of the three migration categories that we define (breeding, refuge, and tracking) are associated with particular life‐history strategies. However, the relatively small sample sizes of definitively identifiable migrants of each type, combined with the strong phylogenetic conservatism of movement‐type (all flying mammals are bats, the overwhelming majority of almost‐exclusively swimming mammals are cetaceans) made fitting comparative models to our data challenging. We therefore take a dual approach: examining the influence of locomotion (walking, swimming, and flying) and the influence of life‐history variables.

### 2.7 The distribution of migration throughout the mammal phylogeny: phylogenetic dispersion (1)

Phylogenetic signal reflects whether species’ traits are randomly distributed throughout a phylogeny (Blomberg, Garland, & Ives, 2003; Freckleton et al., 2002). Its measurement for binary traits (such as migration) is often considered more challenging than for continuous traits (e.g., body mass); here we use *D* to quantify phylogenetic signal (Fritz & Purvis, 2010), which is based on Felsenstein's (2005) Brownian threshold model of character evolution. Values of *D* <1 indicate traits whose distribution across the phylogeny is more phylogenetically restricted than would be expected under a random distribution of traits across the phylogeny, whereas a value <0 indicates traits more restricted than expected under a Brownian threshold model (Felsenstein, 2005). Thus, *D* values <1 are consistent with a moderate degree of phylogenetic signal (i.e., nonrandom distribution of traits within a phylogeny), and values <0 (i.e., negative *D* values) are consistent with a very strong degree of phylogenetic signal. There is some controversy in the literature as to what constitutes a strong degree of phylogenetic signal (e.g.,(Losos, 2011)); by referencing a specific model of trait evolution (Brownian) we are making our opinions explicit. We used the function *phylo.d* in the R package *caper* (Orme et al., 2012) to calculate *D*.

In Table S2 in Appendix S1, we report *D* values calculated across the 1,000 mammal phylogenies. The first column, “migration: overall,” describes the distribution of migratory mammals (*n *=* *158) in the phylogeny of all mammals (*n *=* *965) for which we were able both to obtain movement data and to place within the phylogeny (Fig. S1c in Appendix S1). The second, third, and fourth columns report the distribution of breeding (*n *=* *24), refuge (*n *=* *95), and tracking (*n *=* *59) migrants, respectively, in the phylogeny of all mammals for which we were able to determine migration category.

### 2.8 Locomotion and migration type (2)

As migration is a special kind of movement, it seems likely to be influenced by locomotion type. By creating a simple contingency table, we found that few walking or flying mammals were breeding migrants, while many were refuge migrants (Table S3 in Appendix S1). However, such a contingency table does not account for the phylogenetic nonindependence of species. These patterns might be driven by a few clades that share locomotion and migratory characteristics.

Thus, to account for the nonindependence of species, we calculated the fraction of shared branch length (*PhyloSor* (Bryant et al., 2008); calculated in *picante* (Kembel et al., 2010)) between each migration and locomotion type across each of our 1000 candidate phylogenies. To facilitate the comparison of values (as the total number of species in each category varies), we report standard effect sizes (SES) of each value (Gurevitch, Morrow, Wallace, & Walsh, 1992). Each SES is defined as:

$$\frac{\text{obs}-{\text{mean}}_{\mathrm{rnd}}}{SD_{\mathrm{rnd}}}$$

where obs is the observed shared branch length, mean_rnd_ the mean shared branch length in 100 null permutations, and *SD*_rnd_ the standard deviation of the shared branch length in the same null permutations. We performed 50 null permutations for each phylogeny, shuffling species’ migration types following a trial‐swap null algorithm (Miklós & Podani, 2004) while keeping species’ locomotion‐type constant. Locomotion was not shuffled because it was assumed to be a relatively inherent and evolutionarily difficult to change trait compared to migration. Such SES values allow us to assess the significance of the difference between observed shared branch length and the null expectation given the distribution of locomotion on the phylogeny. In Table S4 in Appendix S1, we give these results, showing that swimming mammals were most closely associated with breeding migrations, flying mammals (bats) were most closely associated with refuge migrations, and walking mammals were associated with both refuge and tracking migrations.

### 2.9 Migration and life history variables (3)

We were interested in the co‐occurrence of migration with various ecological and life‐history variables. The variables we chose are listed below along with our rationale for their inclusion in this analysis. All variables except for Red List category and locomotion were taken from PanTHERIA (Jones et al., 2009).

- Body mass—The logarithm (base ten) of adult body mass (grams). It has been theorized that larger animals may be more likely to migrate due to energetic constraints (Alexander, 2005), so we expect migrants to be larger than nonmigrants, although this may vary based on migration type.
- Habitat breadth—Number of habitat types (above‐ground dwelling, aquatic, fossorial, and ground dwelling) species were observed within. Since migratory species often travel between and through distinct habitats (Robinson et al., 2009), we expect migrants to have greater habitat breadth than nonmigrants.
- Trophic level—Lower levels indicate herbivores, greater values indicate increased dependence on animal food. Species at higher trophic levels are predators and therefore we expect that those that are migratory would be moving to follow migratory prey (tracking migrants). In contrast we expect species at lower trophic levels to be moving to escape predators (refuge migrants).
- Diet breadth—Number of different dietary categories (vertebrate, invertebrate, fruit, flowers/nectar/pollen, leaves/branches/bark, seeds, grass and roots/tubers) upon which species depended. It has been suggested that migrants have more specialized diets than nonmigrants (e.g., diet specialization hypothesis; Boyle, Conway, & Bronstein, 2011). We expect migrants to have narrower diet breadth than nonmigrants, especially for tracking migrants, which follow specific resources rather than using alternative ones in a single place.
- Red List—Threat assessment categorization according to the IUCN Red List (Mace et al., 2008). We ran our analyses with the Red List category coded in two ways. First, we coded the categories as numbers, as has been performed in past analyses with mammals (see Cardillo et al., 2005; Isaac, Turvey, Collen, Waterman, & Baillie, 2007): 1 (least concern), 2 (near threatened), 3 (vulnerable), 4 (endangered), and 5 (critically endangered). Second, as a more conservative approach, we considered Red List category as a binary factor contrasting nonthreatened species (least concern and near threatened) with threatened species (all others). Migrants are often viewed as both more threatened due to the vulnerabilities of migration, and more resilient due to their ability to exploit multiple areas and resources (Robinson et al., 2009).
- Locomotion—Means of locomotion used for migration (walking, flying, or swimming). Determined as described above.

As with the phylogenetic signal results (above), we present four sets of results; the first describes drivers of migration in the phylogeny of all mammals for which we were able to obtain sufficient life history and migration data (605 mammals; Fig. S1 and Table S5 in Appendix S1). In the following tables, we model the distribution of breeding (*n *=* *18; Table S6 in Appendix S1), refuge (*n *=* *64; Table S7 in Appendix S1), and tracking (*n *=* *46; Table S8 in Appendix S1) migrants, respectively, in the phylogeny of all mammals for which we were able to determine migration category (*n *=* *113). We follow the advice of Cole (2015) for the presentation of coefficients and their associated standard deviations, while for *p*‐values we present at three decimal places following the defaults in the R (R Core Team 2014) statistical software.

Each of these models were fit using phylogenetic logistic regression (Ho & Ané, 2014) to all of our candidate phylogenies, and the average results across all of them are presented. We choose to report the averaged results of a single model tested across multiple phylogenies, as opposed to attempting AIC‐based model simplification across all phylogenies. We argue such an approach should, if anything, be conservative (see Whittingham, Stephens, Bradbury, & Freckleton, 2006 for more discussion), and circumvents the issues of (1) averaging across models whose estimates of phylogenetic signal differ and (2) comparing candidate models based on different underlying data (the changing phylogeny itself). We attempted, but do not present here, two additional methods of modeling migration: phylogenetic generalized least squares (PGLS; Freckleton et al., 2002; Orme et al., 2012) and generalized estimating equations (GEE; Paradis & Claude, 2002; Paradis, Claude, & Strimmer, 2004). In the case of PGLS, our models explained an extremely low proportion of variation (*r* ^2^ < 5%); as they were never intended to be used with binary data, this is unsurprising. The GEE models had convergence issues; we suggest this is related to the strong phylogenetic structure in our variables (see ‘Phylogenetic dispersion’ above), but note that where models converged their results were qualitatively similar to those we present here.

## 3 RESULTS

**Figure 1:**
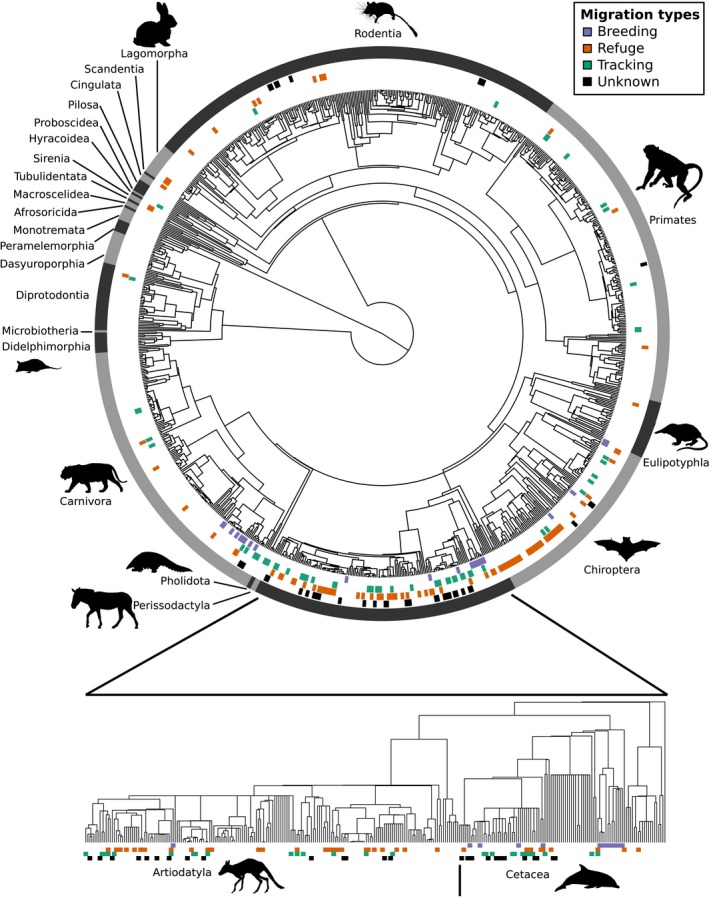
Migration distributed across mammals, but clustered in the Cetartiodactyla. (top) A circular phylogeny of all mammals with definitive migration data (complete phylogeny in Fig. S2 in Appendix S1). Markers around the phylogeny indicate breeding (purple), refuge (orange), and tracking (green) migrants; black markers indicate species whose migration type is unclear; the remaining species are nonmigratory. The outer‐most ring demarcates taxonomic orders. (bottom) The Cetartiodactyla phylogeny is expanded, with labels as above . See Figure 2 for silhouette credits

**Figure 2:**
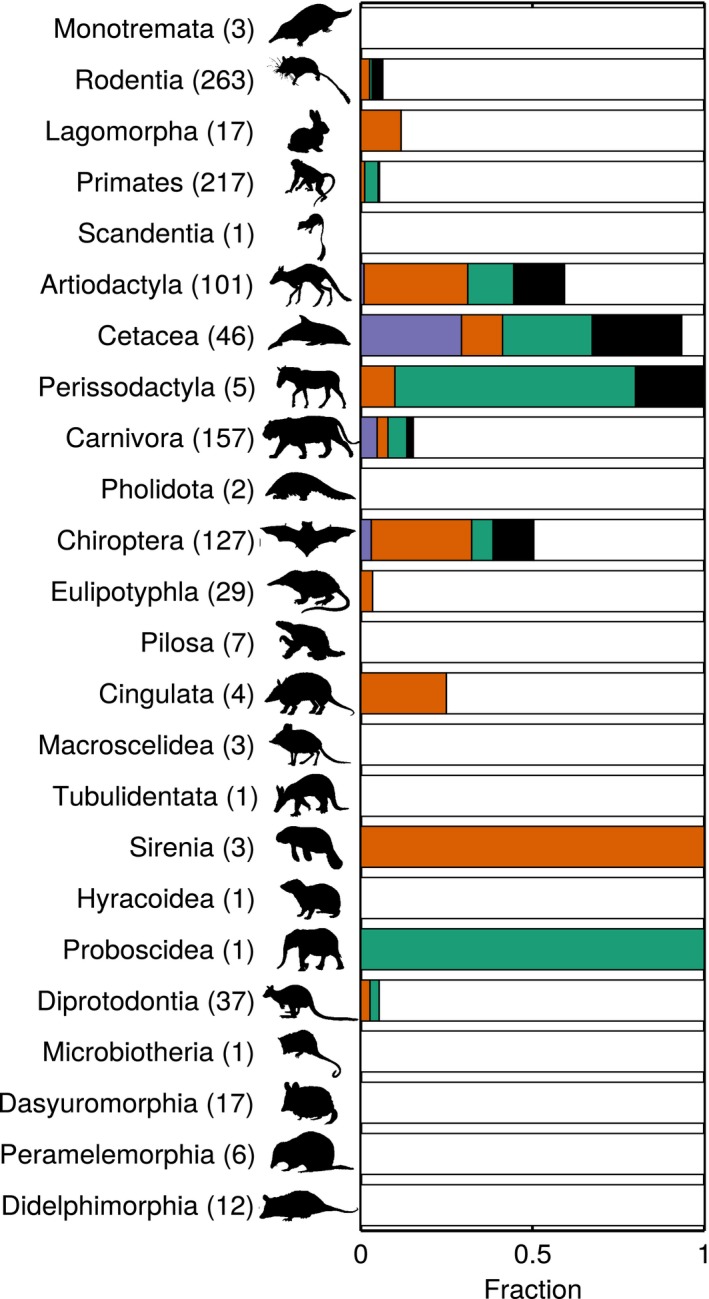
Movement patterns by mammalian order. The fraction of species with known movement patterns that have breeding, refuge, and tracking migrations, an unclear motivation for migration, and no migratory behavior (colors as in Figure 1). Numbers in parentheses indicate the number of species with known movement patterns in each order. The order Cetartiodactyla has been split into Cetacea and Artiodactyla here. Silhouettes are all Public Domain except for: Microbiotheria, Dasyuromorphia, and Didelphimorphia (all Sarah Werning) and Monotremata (Nobu Tamura, vectorized by T. Michael Keesey), which are all CC BY 3.0 (https://creativecommons.org/licenses/by/3.0/)

We analyzed only the 1,062 species whose movement patterns we could definitively classify as migratory or nonmigratory (see ‘*Classifying movement patterns*’ above for criteria). Of these, over a fifth (235) were migratory. These migrants were present across the mammal phylogeny (in 12 of the 27 mammalian orders; Figures 1, 2) including in rodents, rabbits, and primates, clades often overlooked in studies of migration. We classified another 158 mammals as possibly migratory (Fig. S2 in Appendix S1). Although migration was not limited to particular clades, it was not randomly distributed throughout the mammal tree of life (*D *=* *0.42; Table S2 in Appendix S1) and was particularly common in whales, sirenians, and ungulates (Figure 2). In addition to these highly migratory clades, a number of other taxa contained at least a few migratory species.

We were able to infer the factors driving migration for 180 migrant species; these species could all be classified as at least one of the migration types (breeding, refuge, tracking). Of the three types, refuge was the most common (108 species), tracking second (67 species), and breeding was least common (30 species). Each migration type was clustered on the phylogeny, but, like migration generally, was not limited to a single clade (Figure 1; Table S2 in Appendix S1).

To determine whether migrants are associated with specific ecological and life history traits or higher IUCN Red List risk category, we conducted a phylogenetically corrected logistic regression. Both body size and locomotion type differed between migrants and nonmigrants; larger mammals and flying mammals (bats) were more likely to migrate (Table S5 in Appendix S1). However, we found no significant association with Red List category, diet breadth, trophic level, or habitat breadth.

To determine whether the three migration types (refuge, breeding, tracking) were supported by differences in life history traits, we conducted similar regressions within known migratory mammals with sufficient life history data (114 species). Again, body mass was an important factor; breeding migrants were larger, on average (median body mass = 137 kg; IQR = 185), than refuge (3 kg; IQR = 76) or tracking (54 kg; IQR = 195) migrants (*p *=* *0.035; Tables S6, S9 in Appendix S1). Trophic level was also significant: Carnivorous migrants were most likely to have tracking migrations (*p *=* *0.026; Table S8 in Appendix S1). In contrast, diet breadth and habitat breadth were not significant factors. Tracking migrants had higher IUCN Red List risk on average than other migrant types (*p *=* *0.017; Tables S8, S9 in Appendix S1). Each of these relationships with migration type was significant in the regression with Red List as a numeric variable, but was not significant in the more conservative regression with Red List as a binary factor (Tables S6, S8 in Appendix S1).

Primary locomotion type (flying, swimming, walking) was also associated with migration type (Tables S3, S4 in Appendix S1). Correcting for phylogenetic constraint by measuring the fraction of branch length shared by two categories, swimming mammals were most closely associated with breeding migrations (0.483); this was more than expected by random assignment, with a strong standard effect size (SES: 3.118). Flying mammals were most closely associated with refuge migrations (0.397, SES: 1.338), and walking mammals were associated with both refuge (0.248, SES: 0.578) and tracking (0.201, SES: 0.802) migrations (Table S4 in Appendix S1).

## 4 DISCUSSION

Here we have presented the first comprehensive study of migratory behavior across all extant mammal species for which sufficient data exists for analysis. We also group migratory species into three types (refuge, breeding, tracking) based on the main ecological factors driving their movement. Our findings of significant phylogenetic signal suggest that migration and migration type are both conserved across the mammal tree of life. We show that although migratory species are particularly common within certain lineages, they are present in about half of all mammalian orders. We find that body size, locomotion type, and diet are associated with different migration types and that one type (tracking) is associated with higher risk under the IUCN Red List categorization. Together, these results indicate that our proposed categorization scheme represents real differences in migratory species and that we may be better able to predict the response and viability of migratory species under changing conditions (including ecosystem composition, climate, and seasonality) by considering migration in terms of these three types.

The presence of migrants in so many clades within mammals indicates a need for further study of movement patterns in all species, not just known migrants. These distinct occurrences merit further study as they potentially represent independent and unique origins of migration. The distribution pattern we observed (Figure 1) provides only partial support for the hypothesis that migration is not phylogenetically constrained (Alerstam et al., 2003). Given the mixture of migratory states both among and within lineages, it is possible that two separate factors play a role in whether a species migrates: first, an evolved capacity for migration (which may include cognitive and navigational abilities) and second, current ecological conditions (which may determine whether species within capable lineages actually do migrate). For example, it seems likely that cetaceans evolved the necessary abilities for migration early on, but while some (e.g., sperm whale, *Physeter macrocephalus*; Whitehead, 1996) occupy niches that favor migration, others do not (e.g., dwarf sperm whale, *Kogia sima;* Culik, 2004).

Each specific migration type (breeding, refuge, tracking, as defined based on the primary factors driving migration) was, like migration generally, not limited to a single clade of mammals. Within the Cetartiodactyla (the clade with the greatest number of migrants), the three migration types were mixed throughout. This indicates that these three types may not require the evolution of distinct migratory capabilities, rather, migration as an ability may have evolved once historically and then was commandeered for different ecological needs. Furthermore, these types are not mutually exclusive: 25 species had migrations that were classified as two types. For example, the northern elephant seal (*Mirounga angustirostris*) has two distinct migrations per year: one refuge and one breeding (Stewart & Delong, 1995).

We initially expected that migrants would be at higher risk because they have needs that must be met in both locations and they must travel between locations (Robinson et al., 2009). However, IUCN Red List category was not a significant factor in predicting migratory behavior overall. Despite this, IUCN Red List category did predict migration type within migrants, suggesting that tracking migrants may be more susceptible to environmental change and disturbance. The lack of association with specific ecological characteristics indicates that migrants are not necessarily more or less specialized than nonmigrants. Thus, we did not find support here for the ideas (suggested for other species) that migrants, as a whole, have either particularly broad (Robinson et al., 2009) or narrow (Hardy, Griffin, Kuenzi, & Morrison, 2004) habitat breadth or more specialized (Boyle et al., 2011) or generalized (Dennis, Dapporto, Fattorini, & Cook, 2011) diets.

Finally, we found that several traits were each associated with specific migration types. Breeding migrants had larger body sizes, which may enable them to energetically sustain both the travel between sites and, often, a time of complete or partial fasting while they are in the breeding habitat. Refuge migrants, which move to escape unfavorable conditions, were smaller than the other types; consistent with the observation that small animals are more susceptible to extreme seasonality (Lindstedt & Sisterson, 1985). Many carnivorous migrants had tracking migrations, likely because many follow a prey species that is itself migratory (e.g., cheetahs that follow migratory gazelles; Durant, Caro, Collins, Alawi, & FitzGibbon, 1988). The associations between locomotion type and migration type suggest that locomotion‐related energetic may constrain the type of migration that any given species can evolve. Future studies aimed at gaining a deeper understanding of these connections would improve predictions of how migration will respond in the face of changing environmental conditions.

## 5 CONCLUSIONS

Mammals—across many different clades—migrate seasonally to breed, find refuge, or track food resources. Our results show that larger mammals and those that fly, that is, bats, are particularly likely to migrate. Within migrants, body mass, trophic level, and locomotion are each associated with at least one of the three migration types (breeding, refuge, tracking). Migratory species’ abilities to adapt to changing conditions, and thus their extinction risks, are likely to be influenced by the specific factors captured in these three categories. In particular, tracking migrants are at higher risk of extinction that other migrant types, suggesting that they may be more susceptible to changing conditions, including climate change and human disturbance. Future research and conservation efforts should therefore consider how migration type (in addition to overall migration tendency) interacts with both conservation threats and efforts.

## CONFLICT OF INTEREST

None declared.

## AUTHOR CONTRIBUTIONS

AKS conceived the study. GEG designed the data collection methods. GEG and AKS compiled the database, and WDP conducted the statistical analyses. All authors discussed the results, analysis, and interpretation. GEG and AKS wrote the manuscript, with contributions from WDP.

## DATA ACCESSIBILITY

The migration data we collected as well as the scripts used to conduct our analyses are available from Dryad (Gnanadesikan, Pearse, & Shaw, 2017).
