# Supplementary material for: Evolution of mammalian migrations for refuge, breeding, and food
Source: Ecol Evol. 2017 Jun 22;7(15):5891–900. doi: 10.1002/ece3.3120 (PMC5551087; doi:10.1002/ece3.3120)
Supplement: Supplementary file 1 [file ECE3-7-5891-s001.pdf]

Supporting Information

Figure S1. An overview of the sample sizes used for each analysis; the area of each circle is proportional to the number of species in that category. (A) The full list of extant mammals included in the database (5420, grey), the subset that were categorized as definitively migratory or non-migratory (1062, black), and the subset of those that were migratory (235, red). (B) Of those 235 migrants, 180 were classified as breeding (B, purple), refuge (R, orange), tracking (T, green), or a combination thereof; 25 species were classified as two types and included in analyses for each type. (C) The subset of species (965) with both definitive movement and phylogenetic data; of those, 158 were migrants. (D) The subset of species (605) with both definitive movement and life history data; of those, 113 were migrants.

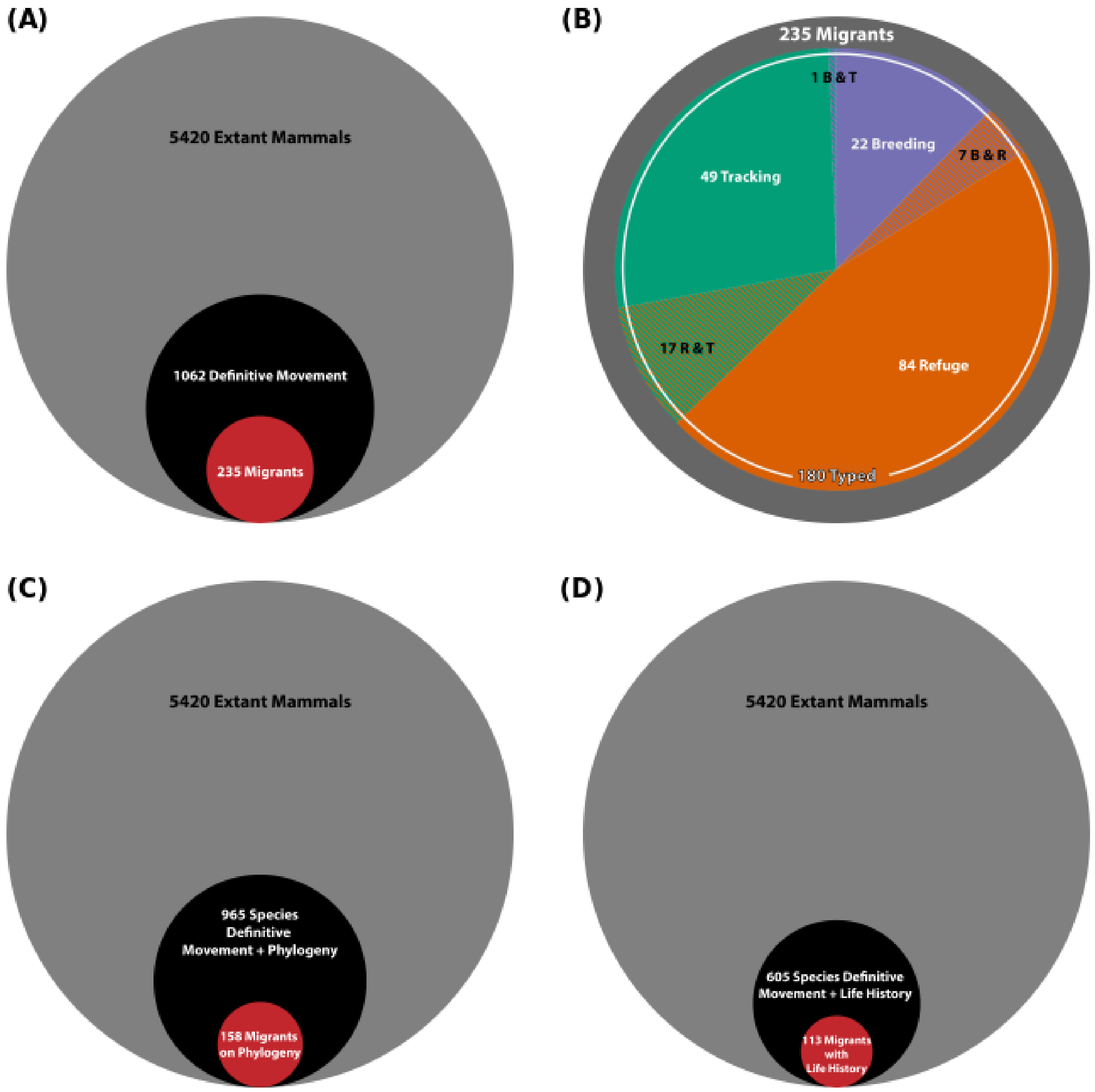

Figure S2. A circular phylogeny of all mammals. Tree branches colors correspond to knowledge of movement patterns: definitively migratory or non-migratory (black; shown in Fig. 1), unclear movement (grey), and data deficient (red). Markers around the outside of the phylogeny indicate breeding (purple), refuge (orange), and tracking (green) migrants, whereas black markers indicate species definitely migrating whose categorization is unclear due to a lack of data. The outer-most ring indicates broad taxonomic groups within the mammals.

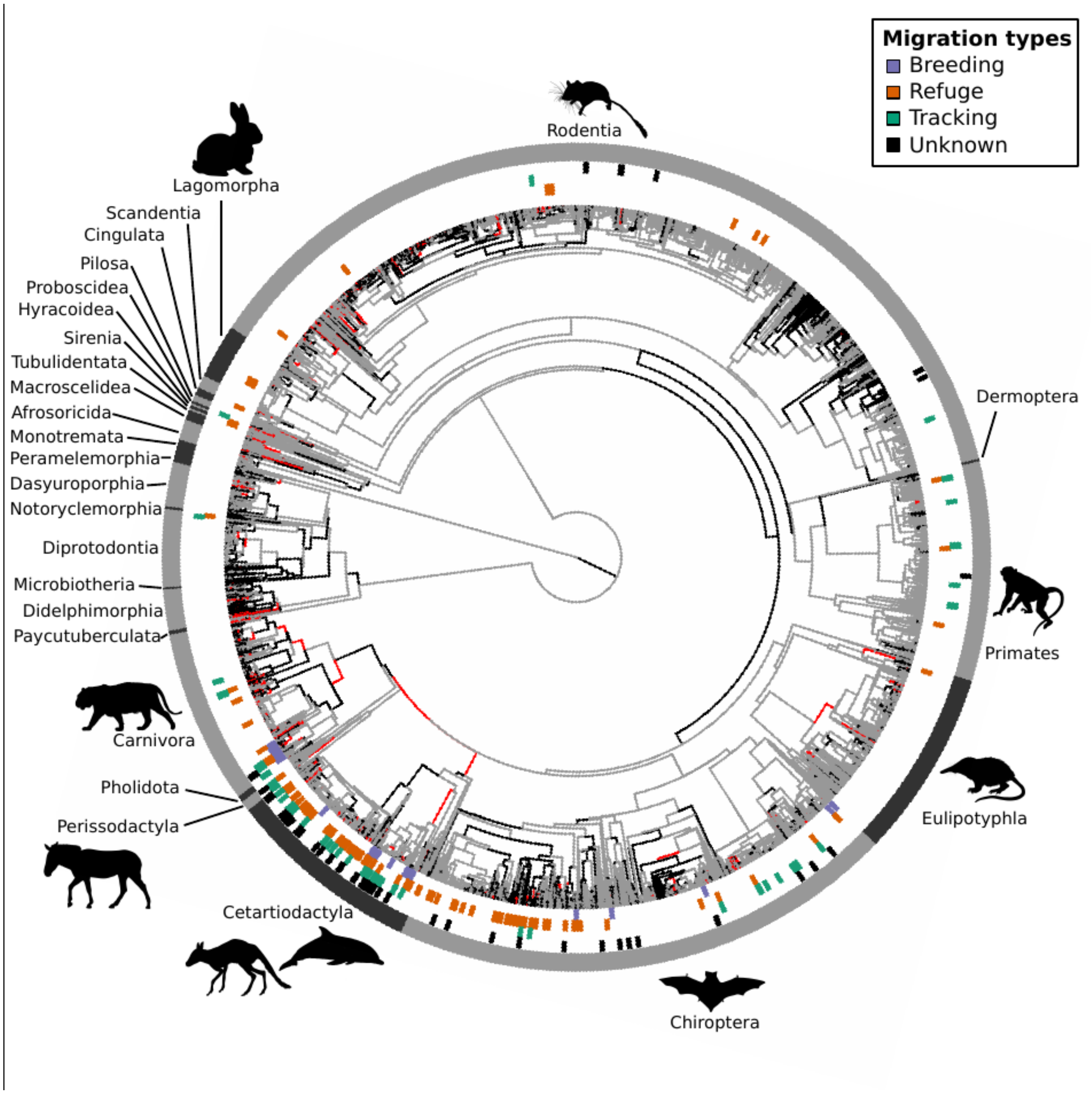

Table S1. The order, family, genus and species of all (235) migratory species considered in our analysis, along with their migration type (B=breeding, R=refuge, T=tracking, R=refuge, U=unknown type), and the ID numbers of the references used to make these classifications (references listed at end). See Dryad repository (DOI: XXX) for data on all mammals species.

Order	Family	Genus	Species	Type	Ref ID
CARNIVORA	CANIDAE	*Canis*	*lupus*	T	537; 3642
	CANIDAE	*Cerdocyon*	*thous*	R	3497
	FELIDAE	*Acinonyx*	*jubatus*	T	3463; 3464
	FELIDAE	*Panthera*	*uncia*	RT	1230; 3485
	FELIDAE	*Puma*	*concolor*	T	100; 529
	MUSTELIDAE	*Pteronura*	*brasiliensis*	R	3461
	ODOBENIDAE	*Odobenus*	*rosmarus*	BR	40; 60; 72
	OTARIIDAE	*Callorhinus*	*ursinus*	B	38
	OTARIIDAE	*Eumetopias*	*jubatus*	B	39
	OTARIIDAE	*Neophoca*	*cinerea*	B	41; 42
	OTARIIDAE	*Zalophus*	*californianus*	B	45
	PHOCIDAE	*Cystophora*	*cristata*	BR	49
	PHOCIDAE	*Histriophoca*	*fasciata*	U	58
	PHOCIDAE	*Lobodon*	*carcinophaga*	RT	56; 3645
	PHOCIDAE	*Mirounga*	*angustirostris*	BR	57
	PHOCIDAE	*Mirounga*	*leonina*	T	65
	PHOCIDAE	*Ommatophoca*	*rossii*	BR	67
	PHOCIDAE	*Pagophilus*	*groenlandicus*	BT	54; 55
	PHOCIDAE	*Phoca*	*largha*	U	68; 69
	PHOCIDAE	*Pusa*	*caspica*	B	40; 50
	PHOCIDAE	*Pusa*	*sibirica*	U	40; 53
	URSIDAE	*Ursus*	*arctos*	T	40; 100
	URSIDAE	*Ursus*	*maritimus*	T	100; 511; 512
	URSIDAE	*Ursus*	*thibetanus*	T	1231
CETARTIODACTYLA	ANTILOCAPRIDAE	*Antilocapra*	*americana*	R	1208
	BALAENIDAE	*Balaena*	*mysticetus*	RT	6; 14; 16
	BALAENIDAE	*Eubalaena*	*australis*	B	7; 8; 13
	BALAENIDAE	*Eubalaena*	*glacialis*	B	13
	BALAENIDAE	*Eubalaena*	*japonica*	B	13
	BALAENOPTERIDAE	*Balaenoptera*	*acutorostrata*	B	6; 37
	BALAENOPTERIDAE	*Balaenoptera*	*bonaerensis*	B	6; 11
	BALAENOPTERIDAE	*Balaenoptera*	*borealis*	B	5; 6

CETARTIODACTYLA	BALAENOPTERIDAE	*Balaenoptera*	*musculus*	B	1; 2; 356; 358
	BALAENOPTERIDAE	*Balaenoptera*	*physalus*	B	1; 6
	BALAENOPTERIDAE	*Megaptera*	*novaeangliae*	B	3; 4; 6; 9; 10; 16
	BOVIDAE	*Addax*	*nasomaculatus*	T	1206
	BOVIDAE	*Alcelaphus*	*buselaphus*	U	186; 3259
	BOVIDAE	*Ammotragus*	*lervia*	U	40; 1221
	BOVIDAE	*Bison*	*bison*	RT	1209
	BOVIDAE	*Bos*	*javanicus*	T	40
	BOVIDAE	*Bos*	*mutus*	U	1235
	BOVIDAE	*Budorcas*	*taxicolor*	T	3304
	BOVIDAE	*Capra*	*cylindricornis*	R	3307
	BOVIDAE	*Capra*	*falconeri*	R	40; 3654
	BOVIDAE	*Capra*	*sibirica*	R	1210
	BOVIDAE	*Connochaetes*	*gnou*	T	186; 1205
	BOVIDAE	*Connochaetes*	*taurinus*	T	186; 1203
	BOVIDAE	*Damaliscus*	*lunatus*	RT	186
	BOVIDAE	*Eudorcas*	*thomsonii*	RT	40
	BOVIDAE	*Gazella*	*cuvieri*	U	40; 1222
	BOVIDAE	*Gazella*	*dorcas*	T	1211
	BOVIDAE	*Gazella*	*gazella*	T	40; 3321
	BOVIDAE	*Gazella*	*subgutturosa*	R	1212
	BOVIDAE	*Hemitragus*	*jemlahicus*	U	40; 1223
	BOVIDAE	*Kobus*	*kob*	R	3327; 3328
	BOVIDAE	*Kobus*	*leche*	R	186
	BOVIDAE	*Naemorhedus*	*goral*	U	40; 3370; 3643
	BOVIDAE	*Nanger*	*granti*	U	1226
	BOVIDAE	*Oreamnos*	*americanus*	RT	1215
	BOVIDAE	*Oryx*	*gazella*	U	186; 3259
	BOVIDAE	*Ovibos*	*moschatus*	U	3380; 3646
	BOVIDAE	*Ovis*	*ammon*	R	1216
	BOVIDAE	*Ovis*	*canadensis*	R	1217
	BOVIDAE	*Ovis*	*dalli*	R	1218
	BOVIDAE	*Pantholops*	*hodgsonii*	B	1219
	BOVIDAE	*Procapra*	*gutturosa*	T	1201
	BOVIDAE	*Pseudois*	*nayaur*	R	3391; 3392
	BOVIDAE	*Rupicapra*	*pyrenaica*	R	3273

CETARTIODACTYLA	BOVIDAE	*Rupicapra*	*rupicapra*	U	3259; 3274
	BOVIDAE	*Saiga*	*tatarica*	R	1200
	BOVIDAE	*Syncerus*	*caffer*	U	186
	BOVIDAE	*Tragelaphus*	*oryx*	T	186
	BOVIDAE	*Tragelaphus*	*scriptus*	R	186
	BOVIDAE	*Tragelaphus*	*spekii*	R	186
	CAMELIDAE	*Camelus*	*ferus*	R	3305; 3653
	CAMELIDAE	*Lama*	*guanicoe*	R	3330
	CERVIDAE	*Alces*	*alces*	R	1207
	CERVIDAE	*Alces*	*americanus*	T	3260
	CERVIDAE	*Axis*	*porcinus*	R	3267
	CERVIDAE	*Blastocerus*	*dichotomus*	R	3297
	CERVIDAE	*Capreolus*	*capreolus*	R	3272
	CERVIDAE	*Capreolus*	*pygargus*	R	1202
	CERVIDAE	*Cervus*	*elaphus*	U	40
	CERVIDAE	*Cervus*	*nippon*	R	3312
	CERVIDAE	*Dama*	*dama*	R	186; 3314
	CERVIDAE	*Hippocamelus*	*antisensis*	U	40
	CERVIDAE	*Hippocamelus*	*bisulcus*	U	1224
	CERVIDAE	*Odocoileus*	*hemionus*	RT	1213
	CERVIDAE	*Odocoileus*	*virginianus*	R	1214
	CERVIDAE	*Przewalskium*	*albirostris*	U	3390
	CERVIDAE	*Rangifer*	*tarandus*	R	40; 1220
	CERVIDAE	*Rucervus*	*eldii*	T	1228
	DELPHINIDAE	*Cephalorhynchus*	*commersonii*	U	26; 33
	DELPHINIDAE	*Delphinus*	*delphis*	T	21; 33
	DELPHINIDAE	*Globicephala*	*macrorhynchus*	T	19; 33
	DELPHINIDAE	*Globicephala*	*melas*	T	19; 33
	DELPHINIDAE	*Grampus*	*griseus*	B	33
	DELPHINIDAE	*Lagenorhynchus*	*acutus*	T	33
	DELPHINIDAE	*Lagenorhynchus*	*albirostris*	U	33
	DELPHINIDAE	*Lagenorhynchus*	*australis*	B	33
	DELPHINIDAE	*Lagenorhynchus*	*obliquidens*	T	33
	DELPHINIDAE	*Lagenorhynchus*	*obscurus*	T	33
	DELPHINIDAE	*Lissodelphis*	*borealis*	U	33
	DELPHINIDAE	*Orcaella*	*brevirostris*	RT	33
	DELPHINIDAE	*Orcinus*	*orca*	T	22; 23; 24; 25; 33

CETARTIODACTYLA	DELPHINIDAE	*Sotalia*	*fluviatilis*	R	33; 3649
	DELPHINIDAE	*Sousa*	*chinensis*	T	33; 3651
	DELPHINIDAE	*Sousa*	*teuszii*	U	33
	DELPHINIDAE	*Stenella*	*attenuata*	U	33
	DELPHINIDAE	*Stenella*	*coeruleoalba*	U	33
	DELPHINIDAE	*Stenella*	*longirostris*	U	33
	DELPHINIDAE	*Tursiops*	*truncatus*	U	27; 33
	ESCHRICHTIIDAE	*Eschrichtius*	*robustus*	B	6; 16
	HIPPOPOTAMIDAE	*Hippopotamus*	*amphibius*	R	186
	INIIDAE	*Inia*	*geoffrensis*	T	28
	MONODONTIDAE	*Delphinapterus*	*leucas*	R	6; 17; 533
	MONODONTIDAE	*Monodon*	*monoceros*	BR	16; 20; 33
	PHOCOENIDAE	*Neophocaena*	*phocaenoides*	U	33
	PHOCOENIDAE	*Phocoena*	*phocoena*	R	16; 29; 33
	PHOCOENIDAE	*Phocoena*	*spinipinnis*	U	33
	PHOCOENIDAE	*Phocoenoides*	*dalli*	B	30; 33
	PHYSETERIDAE	*Physeter*	*macrocephalus*	T	6; 15; 16
	PLATANISTIDAE	*Platanista*	*gangetica*	T	6; 40
	SUIDAE	*Sus*	*barbatus*	R	40; 1233
	ZIPHIIDAE	*Berardius*	*arnuxii*	R	32; 33
	ZIPHIIDAE	*Berardius*	*bairdii*	U	33
	ZIPHIIDAE	*Hyperoodon*	*ampullatus*	U	6; 18; 33
CHIROPTERA	EMBALLONURIDAE	*Diclidurus*	*albus*	U	189; 191; 199; 319; 3644
	EMBALLONURIDAE	*Taphozous*	*melanopogon*	B	145; 175
	EMBALLONURIDAE	*Taphozous*	*nudiventris*	U	132; 145
	HIPPOSIDERIDAE	*Asellia*	*tridens*	U	132; 145
	HIPPOSIDERIDAE	*Hipposideros*	*armiger*	U	145; 200; 208; 216; 222
	HIPPOSIDERIDAE	*Hipposideros*	*commersoni*	RT	145; 167; 235; 3648
	HIPPOSIDERIDAE	*Hipposideros*	*lankadiva*	U	145; 216; 239; 251
	MOLOSSIDAE	*Nyctinomops*	*macrotis*	R	100; 189; 191
	MOLOSSIDAE	*Tadarida*	*australis*	RT	134; 257
	MOLOSSIDAE	*Tadarida*	*brasiliensis*	U	100; 106; 120

CHIROPTERA	NATALIDAE	*Natalus*	*stramineus*	R	314; 356
	NYCTERIDAE	*Nycteris*	*thebaica*	U	102; 186; 206
	PHYLLOSTOMIDAE	*Choeronycteris*	*mexicana*	RT	100
	PHYLLOSTOMIDAE	*Leptonycteris*	*curasoae*	B	361; 362
	PHYLLOSTOMIDAE	*Leptonycteris*	*nivalis*	U	100; 120; 363
	PHYLLOSTOMIDAE	*Leptonycteris*	*yerbabuenae*	U	100; 101; 364; 387
	PHYLLOSTOMIDAE	*Sturnira*	*lilium*	R	189; 191; 356; 404
	PTEROPODIDAE	*Eidolon*	*helvum*	T	102; 103; 107; 108; 145; 216
	PTEROPODIDAE	*Myonycteris*	*torquata*	T	102; 103; 112; 113; 186
	PTEROPODIDAE	*Nanonycteris*	*veldkampii*	T	145; 153
	PTEROPODIDAE	*Pteropus*	*conspicillatus*	T	103; 134; 136; 186
	PTEROPODIDAE	*Pteropus*	*poliocephalus*	T	103; 134; 137; 138; 145
	PTEROPODIDAE	*Pteropus*	*scapulatus*	U	134; 139; 145
	PTEROPODIDAE	*Rousettus*	*aegyptiacus*	RT	103; 109; 145
	RHINOLOPHIDAE	*Rhinolophus*	*euryale*	BR	145; 217; 250
	RHINOLOPHIDAE	*Rhinolophus*	*mehelyi*	R	145; 217
	RHINOPOMATIDAE	*Rhinopoma*	*microphyllum*	B	132; 170; 175; 182; 185; 207
	VESPERTILIONIDAE	*Antrozous*	*pallidus*	R	100
	VESPERTILIONIDAE	*Chalinolobus*	*morio*	R	134
	VESPERTILIONIDAE	*Eptesicus*	*fuscus*	R	100; 104; 189; 191; 356
	VESPERTILIONIDAE	*Eptesicus*	*nasutus*	U	132; 420
	VESPERTILIONIDAE	*Lasionycteris*	*noctivagans*	R	100; 120
	VESPERTILIONIDAE	*Lasiurus*	*blossevillii*	R	105; 120; 356; 434

CHIROPTERA	VESPERTILIONIDAE	*Lasiurus*	*borealis*	R	100; 435
	VESPERTILIONIDAE	*Lasiurus*	*cinereus*	R	22; 33; 100; 189; 190; 191
	VESPERTILIONIDAE	*Lasiurus*	*intermedius*	U	100
	VESPERTILIONIDAE	*Miniopterus*	*fuliginosus*	U	200; 208; 420; 455
	VESPERTILIONIDAE	*Miniopterus*	*natalensis*	R	102
	VESPERTILIONIDAE	*Miniopterus*	*oceanensis*	BR	134; 161; 243; 267
	VESPERTILIONIDAE	*Miniopterus*	*schreibersii*	R	250; 288; 454
	VESPERTILIONIDAE	*Myotis*	*auriculus*	R	100; 356
	VESPERTILIONIDAE	*Myotis*	*austroriparius*	R	100; 3655
	VESPERTILIONIDAE	*Myotis*	*blythii*	U	208; 288; 420
	VESPERTILIONIDAE	*Myotis*	*brandtii*	R	208; 288
	VESPERTILIONIDAE	*Myotis*	*capaccinii*	R	288
	VESPERTILIONIDAE	*Myotis*	*dasycneme*	R	208; 288
	VESPERTILIONIDAE	*Myotis*	*daubentonii*	R	208; 250; 288
	VESPERTILIONIDAE	*Myotis*	*emarginatus*	RT	250; 288
	VESPERTILIONIDAE	*Myotis*	*grisescens*	R	100
	VESPERTILIONIDAE	*Myotis*	*lucifugus*	R	100; 475
	VESPERTILIONIDAE	*Myotis*	*myotis*	RT	250; 288
	VESPERTILIONIDAE	*Myotis*	*mystacinus*	R	250; 288
	VESPERTILIONIDAE	*Myotis*	*nattereri*	R	288
	VESPERTILIONIDAE	*Myotis*	*septentrionalis*	R	100; 3657
	VESPERTILIONIDAE	*Myotis*	*sodalis*	R	100; 418; 510
	VESPERTILIONIDAE	*Myotis*	*velifer*	R	100; 356
	VESPERTILIONIDAE	*Nyctalus*	*lasiopterus*	U	288
	VESPERTILIONIDAE	*Nyctalus*	*leisleri*	R	213; 214; 250; 288
	VESPERTILIONIDAE	*Nyctalus*	*noctula*	R	200; 208; 288; 420
	VESPERTILIONIDAE	*Nycticeius*	*humeralis*	R	100; 145
	VESPERTILIONIDAE	*Pipistrellus*	*hesperus*	R	100; 120
	VESPERTILIONIDAE	*Pipistrellus*	*nathusii*	R	288; 509
	VESPERTILIONIDAE	*Pipistrellus*	*subflavus*	R	100; 120;

					356
CHIROPTERA	VESPERTILIONIDAE	*Vespertilio*	*murinus*	R	208; 250; 288; 420
CINGULATA	DASYPODIDAE	*Dasypus*	*novemcinctus*	R	1949
DIPROTODONTIA	BURRAMYIDAE	*Burramys*	*parvus*	R	2048
	BURRAMYIDAE	*Cercartetus*	*lepidus*	T	2051
EULIPOTYPHLA	TALPIDAE	*Scapanus*	*townsendii*	R	2846
LAGOMORPHA	LEPORIDAE	*Lepus*	*californicus*	R	2254; 3641
	LEPORIDAE	*Lepus*	*timidus*	R	2249; 3640
PERISSODACTYLA	EQUIDAE	*Equus*	*grevyi*	T	542; 543
	EQUIDAE	*Equus*	*quagga*	T	40; 97; 98; 99; 538; 539; 543
	EQUIDAE	*Equus*	*zebra*	T	40; 538; 543
	RHINOCEROTIDAE	*Ceratotherium*	*simum*	RT	40; 92; 3647
	RHINOCEROTIDAE	*Dicerorhinus*	*sumatrensis*	U	40
PRIMATES	ATELIDAE	*Lagothrix*	*cana*	T	3074
	CEBIDAE	*Cebus*	*albifrons*	U	3046; 3074
	CERCOPITHECIDAE	*Macaca*	*fuscata*	T	3162; 3209
	CERCOPITHECIDAE	*Rhinopithecus*	*bieti*	RT	3210
	CERCOPITHECIDAE	*Rhinopithecus*	*roxellana*	T	3208
	HOMINIDAE	*Gorilla*	*beringei*	T	1232; 2878
	HOMINIDAE	*Homo*	*sapiens*	R	3658
	HOMINIDAE	*Pongo*	*pygmaeus*	T	3254
	LEMURIDAE	*Eulemur*	*fulvus*	R	2929
	LEMURIDAE	*Eulemur*	*rufifrons*	T	2933
	PITHECIIDAE	*Cacajao*	*calvus*	T	3075; 3076
	PITHECIIDAE	*Cacajao*	*melanocephalus*	T	3075
PROBOSCIDEA	ELEPHANTIDAE	*Loxodonta*	*africana*	T	2185; 2186
RODENTIA	CRICETIDAE	*Eolagurus*	*luteus*	T	208
	CRICETIDAE	*Lemmus*	*lemmus*	U	217
	CRICETIDAE	*Lemmus*	*sibiricus*	U	1408
	CRICETIDAE	*Microtus*	*fortis*	R	208; 1446
	CRICETIDAE	*Microtus*	*montanus*	R	1027; 1429
	CRICETIDAE	*Peromyscus*	*maniculatus*	R	668; 1027

RODENTIA	HETEROMYIDAE	*Dipodomys*	*ordii*	R	668; 1027
	MURIDAE	*Apodemus*	*flavicollis*	U	217; 779
	MURIDAE	*Apodemus*	*sylvaticus*	U	217; 778; 779
	MURIDAE	*Meriones*	*libycus*	U	208
	MURIDAE	*Micromys*	*minutus*	R	200; 208; 1251
	MURIDAE	*Otomys*	*angoniensis*	U	186; 1005
	MURIDAE	*Rattus*	*colletti*	R	134; 1253
	MURIDAE	*Rattus*	*rattus*	R	190; 1254
	SCIURIDAE	*Sciurus*	*arizonensis*	U	1741
	SCIURIDAE	*Sciurus*	*niger*	U	668; 1748
	SCIURIDAE	*Tamias*	*dorsalis*	T	1114
SIRENIA	DUGONGIDAE	*Dugong*	*dugon*	R	75; 78; 79
	TRICHECHIDAE	*Trichechus*	*inunguis*	R	75; 76
	TRICHECHIDAE	*Trichechus*	*manatus*	R	75

References

1. Mackintosh, N. A. 1966. The distribution of southern blue and fin whales. In Whales, dolphins, and porpoises, ed. K. S. Norris. 125-144. Univ. Calif. Press, Berkeley.
2. Bailey et al. Beahvioural estimation of blue whale movements in the Northeast Pacific from state-space model analysis of satellite tracks. Endangered Species Research. 2009.
3. Ramussen et al. Southern Hemisphere humpback whales wintering off Central America: insights from water temperature into the longest mammal migration. Biology Letters. 2007. http://www.topp.org/sites/topp.org/files/topp/publications/rasmussen2007.pdf
4. Dawbin, Willaim H. The seasonal Migratory Cycle of Humpback Whales. In Whales, dolphins, and porpoises, ed. Kenneth S. Norris. 1966. 145-?. Univ. Calif. Press, Berkeley.
5. Mizroch, Rice, Breiwick. The Sei Whale Balaenoptera borealis. http://spo.nmfs.noaa.gov/mfr464/mfr4646.pdf
6. Lockyer, C. H. and S. G. Brown. "The migration of whales" in Animal Migration by David J Aidley. Society for Experimental Biology. 1981.
7. Best, et al. Long-range movements of South Atlantic Right Whales Eubalaena Australis.
8. Corkeron, Peter J., Richard C. Connor. Why do baleen whales migrate? Marine Mammal Science 15(4):1228-1245 (October 1999). Society for Marine Mammalogy
9. Brown, et al. Evidence for a Sex-Segregated Migration in the Humpback Whale (Megaptera novaeangliae). The Royal Society. 1995.
10. Horton et al. Straight as an arrow: humpback whales swim constant course tracks during long-distance migration. Biology Letters. April 20 2011.
11. Kasamatsu, Nishiwake, and Ishikawa. Breeding areas and southbound migrations of southern minke whales Balaenoptera acutostrata. Marine Ecology Progress Series. Vol. 119:1-10, 1995. March 23, 1995.
13. Braham, Howard W., Dale W. Rice. The Right Whale, Balaena glacialis. Marine Fisheries Review.
14. Reeves, Randall et al. Distribution and Migration of the Bowhead Whale, Balaena mysticetus, in the the Eastern North American Arctic.
15. Whitehead, Hal. Variation in the feeding Success of Sperm Whales: Temporal Scale, Spatial Scale and Relationship to Migrations. Journal of Animal Ecology. Vol. 65, No. 4 (Jul., 1996), pp. 429-438.
16. Reynolds, John E. III, and Sentiel A. Rommel. Biology of Marine Mammals. Smithsonian Inst. P. Washington: 1999.
17. Smith , Thomas G. Distribution and Movements of Belugas, Delphinapterus leucas, in the Canadian High Arctic. Canadian Journal of Fisheries and Aquatic Sciencens, 1994, 51:1653-1663, 10.1139/f94-166.
18. Whitehead, Hal and Sascha K. Hooker. Status of northern bottlenose whales. International Whaling Commission.

19. Mintzer, Vanessa J. et al. Stomach contents of mass-stranded short-finned pilot whales (Globicephala macrorhynchus) from North Carolina. Marine Mammal Science, 24(): 90-301 (April 2008).
20. Heide-Jorgensen, Mads Peter. The migratory behahviour of narwhals (Monodon monoceros). Canadian Journal of Zoology, 2003, 81:1298-1305, 10.1139/z03-117.
21. Evans, W. E. Orientation Behaviour of Delphinids: Radio Telemetric Studies. Annals of the New York Academy of Sciences. Volume 188, Orietation:Sensory Bases, pp 142-160. December 1971.
22. Iniquez, Miguel A. Seasonal distribution of killer whales (Orcinus orca) in Northern Patagonia, Argentina. Aquatic Mammals 2001, 27.2, 154-161.
23. Nichol, Linda M., David M. Shackleton. Seasonal movements and foraging behaviour of northern resident killer whales (Orcinus orca) in relation to the inshore distribution of salmon (Oncorhynchus spp.) in British Columbia. Canadian Journal of Zoology, 1996, 74:983-991, 10.1139/z96-111.
24. Condy, P.R., R. J. van Aarde, and M. N. Bester. The seasonal occurrence and behaviour of Killer whales Orcinus orca, at Marion Island. J. Zoology., Lond. (1978) 184, 449-464.
25. Heyning, John E., Marilyn E. Dahlheim. Orcinus orca. Mammalian Species, No. 304, pp. 1-9. American Society ofMammalogists. 15 Jan 1988.
26. Coscarella, M. A., S. N. Pedraza, E. A. Crespo. Behavior and seasonal variation in the relative abundance of Commerson's dolphin (Cephalorhynchus commersonii) in northern Patagonia, Argentina. J of Ethology. Vol 28, Number 3, 463-470. 2010.
27. Barco, Susan G. et al. Local Abundance and distribution of Bottlenose Dolphins (Tursiops truncatus) in the nearshore waters of Virginia Beach, Virginia. Marine Mammal Science, 15(2):394-408 (April 1999).
28. Best, Robin C., and Vera M. F. da Silva. Inia geoffrensis. Mammalian Species. No. 426, pp1 1-8. 23 April, 1993.
29. Northridge, Simon P., et al. Distribution and relative abundance of harbour porpoises (Phoecoena phocoena L.), white-beaked dolphins (Legenorhynchus albirostrisGray), and minke whales (Balaenoptera acutorostrata Lacepede) around the British Isles. ICES J of Marine Science, 52: 55-66. 1995.
30. Amano, Maso, and Toshiaki Kuramochi. Segregative Migration of Dall's Porpoise (Phocoenoides Dalli) in the Sea of Japan and Sea of Okhotsk. Marine Mammal Science, 8(2):143-151 (April 1992).
32. Sciliano, Salvatore, and Marco Cesar de Oliveria Santos. On the occurrence of the Arnoux's beaked whale (Berardius arnuxii) in Brazil. J of the Marine Biological Association of the UK, (2003), 83: 887-888.
33. Culik, Boris M. Review of Small Cetaceans: Distribution, Behaviour, Migration and Threats. CMS/CULIK 2004.
37. Pastene, Luis A. et al. Radiation and speciataion of pelagic organisms during periods of global warming: the case of the common minke whale, Balaenoptera acutorostrata. Molecular Ecology (2007) 16, 1481-1495.
38. Kenyon, Karl W., and Ford Wilke. Migration of the Northern Fur Seal, Callorhinus ursinus. Journal of Mammalogy. Vol 34, No 1 (Feb, 1953), pp 86-98.
39. Schusterman, R.J. Steller sea lion - Eumetopias jubatus. In: Handbook of Marine Mammals, Vol 1: The Walrus, Sea Lions, Fur Seals and Sea Otters, S.H. Ridgway and R.J. Harrison (Eds), Academic Press, London.
40. Walker, Ernest P. Walker's Mammals of the World. Ed Ronald M. Nowak, John L. Paradiso. 4th Edition, 1983. Vol II.
41. Gales, Nicholas J., et al. Breeding Biology and Movements of Australian Sea-lions, Neophoca cinerea, off the West Coast of Western Australia. Wildl. Res., 1992, 19, 405-16.
42. Gales, N. J., P.D. Shaughnessy, and T.E. Dennis. Distribution, abundance and breeding cycle of the Australian sea lion Neophoca cinerea (Mammalia: Pinnipedia). J of Zoology, Vol. 234, Issue 3, pp 353–370, November 1994.
45. Aurioles, David, et al. Winter Migration of Subadult Male California Sea Lions (Zalophus Californianus) in the Southern Part of Baja California. J. Mamm. 63(3):513-518, 1983.
49. Andersen, J. M., et al. Movement Patterns of Hooded Seals (Cystophora cristata) in the Northwest Atlantic Ocean During the Post-Moult and Pre-Breed Seasons. J. Northw. Atl. Fish Sci., 42: 1–11. 2009.
50. Härkönen, T. 2008. Pusa caspica. In: IUCN 2011. IUCN Red List of Threatened Species. Version 2011.1. <www.iucnredlist.org>. Downloaded on 20 June 2011.
53. Tarasova, E.N., et al. Polychlorinated Dibenzo-P-Dioxins (PCDDs) and Dibenzofurans (PCDFs) in Baikal Seal. Chemosphere, Vol. 34, No. 11, pp. 2419-2427, 1997.
54. Sergeant, D.E. Feeding, Growth, and Productivity of Northwest Atlantic Harp Seals (Pagophilus groenlandicus). J. Fish. Res. Board Can. 30: 17-29.
55. Folkow, Lars P., Erling S. Nordøy, and Arnoldus S. Blix. Distributiona nd diving behaviour of harp seals (Pagophilus groenlandicus) from the Greenland Sea stock. Polar Biol (2004) 27: 281–298.
56. Adam, Peter J. Lobodon carcinophaga. Mammalian Species. No. 772, pp. 1–14, 15 July 2005.

57. Stewart, Brent S. and Robert L. DeLong. Double Migrations of the Northern Elephant Seal, Mirounga angustirostris. Journal of Mammalogy, Vol. 76, No. 1 (Feb., 1995), pp. 196-205.
58. Boveng, P. L., J. L. Bengtson, T. W. Buckley, M. F. Cameron, S. P. Dahle, B. A. Megrey, J. E. Overland, and N. J. Williamson. 2008. Status review of the ribbon seal (Histriophoca fasciata). U.S. Dep. Commer., NOAA Tech. Memo. NMFS-AFSC-191, 115 p.
60. Garlich-Miller, Joel, et al. Status Review of the Pacific Walrus (Odobenus rosmarus divergens). Jan 2011.
65. Field, Iain C., et al. Resource partitioning through oceanic segregation of foraging juvenile southern elephant seals (Mirounga leonina). Oecologia (2005) 142: 127–135.
67. Bliz, Arnoldus Schytte and Erling S. Nordøy. Ross seal (Ommatophoca rossii) annual distribution, diving behaviour, breeding and moulting, off Queen Maud Land, Antarctica. Polar Biol (2007) 30:1449–1458.
68. Lowry, L.F., K.J. Frost, R. Davis, R.S. Suydam, and D.P. DeMaster. 1994. Movements and behavior of satellite-tagged spotted seals (hoca largha) in the Bering and Chukchi seas. U.S. Dep. Commerce NOAA Tech. Memo. NMFS-AFSC-38, 71 p.
69. Lowry, Lloyd F., et al. Movements and behavior of satellite-tagged spotted seals (Phoca largha) in the Bering and Chukchi Seas. Polar Biol (1998) 19: 221-230.
72. Fay, Francis H. Walrus. In Handbook of Marine Mammals. Ed. Sam H. Ridgway and Richard J. Harrison F.R.S. 1981. Vol 1. Academic Press, London.
75. Reynolds III, John E, and Daniel K. Odell. Manatees and Dugongs. New York: Facts on File. 1991.
76. Arraut, E.M., et al. The lesser of two evils: seasonal migrations of Amazonian manatees in the Western Amazon. Journal of Zoology 280 (2010) 247–256.
78. Holley, David K., Ivan R. Lawler, and Nicholas J. Gales. Summer survey of dugong distribution and abundance in Shark Bay reveals additional key habitat area. Wildlife Research, 2006, 33, 243–25.
79. Marsh, H. et al. The Distribution and Abundance of the Dugong in Shark Bay, Western Australia. Wildl. Res., 1994, 21, 149-61.
92. IUCN SSC African Rhino Specialist Group 2008. Ceratotherium simum. In: IUCN 2011. IUCN Red List of Threatened Species. Version 2011.1. <www. iucnredlist.org>. Downloaded on 23 June 2011.
97. Brooks, C.J., and Stephen Harris. Directed movement and orientation across a large natural landscape by zebras, Equus burchelli antiquorum. Animal Behaviour, 76, 2. 2008.
98. Bartlam-Brooks, H.L.A, M.C. Bonyongo, and Stephen Harris. Will reconnecting ecosystems allow long-distance mammal migrations to resume? A case study of a zebra Equus burchelli migration in Botswana. Oryx, 45(2), 210–216. 2010.
99. Hack, M. A., R. East & D. I. Rubenstein (2002). Status and action plan for the Plains zebra (Equus burchelli). Pp 43-57. In Equids: Zebras, Asses, and Horses. Status Survey and Conservation Action Plan. P. D. Moehlman (ed.). IUCN/SSC Equid Specialist Group. IUCN, Gland, Switzerland
100. Wilson, Don E., and Sue Ruff. The Smithsonian Book of North American Mammals. Washington: Smithsonian Inst. P. 1999.
101. Rojas-Martinez, Alberto, et al. Seasonal distribution of the long-nosed bat (Leptonycteris curasoae) in North America: does a generalized migration pattern really exist? Journal of Biogeography, 26, 1065–1077.
102. Monadjem, Ara, et al. Bats of Southern and Central Africa. Johannesburg: Wits UP. 2001.
103. Walker, Ernest P. Walker's Mammals of the World. Ed Ronald M. Nowak, John L. Paradiso. 4th Edition, 1983. Vol I.
104. Neublam, Daniel J., Thomas J. O'Shea, Kenneth R. Wilson. Autumn Migration and Selection of Rock Crevices as Hibernacula by Big Brown Bats in Colorado. Journal of Mammalogy, 87(3):470-479, 2006.
105. Cryan, Paul M. Seasonal Distribution of Migratory Tree Bats (Lasiurus and Lasionycteris) in North America. Journal of Mammalogy, 84(2):579-593, 2003.
106. Villa R., Bernardo, and E. Lendell Cockrum. Migration in the Guano Bat Tadarida brasiliensis mexicana (Saussure). Journal of Mammalogy, Vol. 43, No. 1 (Feb., 1962), pp. 43-64.
107. Richter, H.V., and G.S. Cumming. Food availability and annual migration f the straw-colored fruit bat. Journal of Zoology, 268 (2006) 35–44.
108. Richter, H.V., and G.S. Cumming. First application of satellite telemtry to track African straw-coloured fruit bat migration. Journal of Zoology, 275 (2008) 172–176.
109. Kwiecinski, Gary G., and Thomas A. Griffiths. Rousettus egyptiacus. Mammalian Species, No. 611. 5 May 1999.
112. Mickleburgh, S., Hutson, A.M., Bergmans, W. & Fahr, J. 2008. Myonycteris torquata. In: IUCN 2011. IUCN Red List of Threatened Species. Version 2011.1. <www.iucnredlist.org>. Downloaded on 28 June 2011.
113. Fleming, Theodore H., and Peggy Eby. Ecology of Bat Migration. In Bat Ecology, ed. Thomas H. Kunz and M.

Brock Fenton. Chicago: U Chicago P. 2003.
120. Whitaker Jr., John O. National Audubon Society Field Guide to North American Mammals. New York: Chanticleer P. 1996.
132. Roberts, T.J. The Mammals of Pakistan. New York: Oxford UP. 1997.
134. Van Dyck, Steve, and Ronald Strahan. The Mammals of Australia. Third Edition. London: Reed New Holland. 2008.
136. Richards, G.C. The Spectacled Flying-fox, Pteropus conspicillatus (Chiroptera: Pteropodidae), in north Queensland. I. Roost sites and distribution patterns. Aust. Mammal. 13: 17-24. 1990.
137. Eby, Peggy. Seasonal Movements of Grey-headed Flying-foxes Pteropus poliocephalus (Chiroptera: Pteropodidae), from Two Maternity Camps in Northern New South Wales. Wildl. Res., 1991, 18, 547-59.
138. Tidemann, Christopher R., and John E. Nelson. Long-distance movements of the grey-headed flying fox (Pteropus poliocephalus). J. Zool., Lond. (2004) 263, 141–146.
139. Vardon, Michael J., et al. Seasonal habitat use by flying-foxes, Pteropus alecto and P. scapulatus (Megachiroptera), in monsoonal Australia. J. Zool., Lond. (2001) 253, 523-535.
145. Nowak, Ronald M. Walker's Bats of the World. Baltimore: Johns Hopkins UP. 1991.
153. Thomas, Donald W. The annual migrations of three species of West African fruit bats (Chiroptera: Pteropodidae). Can. J. Zool., Vol. 61, 1983.
161. Flannery, Tim. Mammals of the South-West Pacific & Moluccan Islands. New York: Cornell UP. 1995.
167. Garbutt, Nick. Mammals of Madagascar. New Haven: Yale UP. 1999.
170. Schlitter, Duanne A., and Mazin B. Qumsiyeh. Rhinopoma microphyllum. Mammalian Species. 27 December 1996.
175. Badwaik, N. Seasonal migration of two species of microchiroptera in relation to breeding cycles. Mammalia, vol. 56, iss 2. 287-290. 1992.
182. Levin, E., et al. Warm winter hibernation in warm ambient temperature by the greater mouse-tailed bat (Rhinopoma microphyllum). Israel J or Ecology & Evolution, vol. 56. Pg 90. 2010.
185. Kumar, T. C. Anand. Reproduction in the rat-tailed bat Rhinopoma kinneari. Proceedings of the Zoological Society of London, vol. 147, iss. 2. Pgs 147-155. December 1965.
186. Skinner, John D., and Reay H.N. Smithers. The Mammals of the Southern African Subregion. Pretoria: U of Pretoria. 1990.
189. Eisenberg, John F. Mammals of the Neotropics: Volume 1: Panama, Colobia, Venezuela, Guyana, Suriname, French Guinea. Chicago: U Chicago P. 1989.
190. Redford, Kent H., and John F. Eisenberg. Mammals of the Neotropics: Volume 2:Chile, Argentina, Uruguay, Paraguay. Chicago: U Chicago P. 1992.
191. Redford, Kent H., and John F. Eisenberg. Mammals of the Neotropics: Volume 3: Ecuador, Peru, Bolivia, Brazil. Chicago: U Chicago P. 1999.
199. Ceballos, Gerardo, and Rodrigo A. Medllin. Diclidurus albus. Mammalian Species. No. 316. 30 June 1988.
200. Francis, Charles M. A Guide to the Mammals of Southeast Asia. Princeton: Princeton UP. 2008.
206. Gray, Paul A., M. Brock Fenton, and Victory Van Cakenberghe. Nycteris thebaica. Mammalian Species. No. 612. 5 May 1999.
207. Finn, Frank. Sterndale's Mammalia of India. New Delhi: Cosmos Publications. 2002
208. Smith, Andrew T., and Yan Xie. A Guide to the Mammals of China. Princeton: Princeton UP. 2008.
213. Vaughan, Terry A., and Rosemary P. Vaughan. Seasonality and the Behavior of the African Yellow-Winged Bat. Journal of Mammalogy, Vol. 67, No. 1 (Feb., 1986), pp. 91-102.
214. Vonhof, Maarten J., and Matina C. Kalcounis. Lavia frons. Mammalian Species. No. 614. 5 May 1999.
216. Menon, Vivek. Mammals of India. Princeton: Princeton UP. 2009.
217. Mitchell-Jones, A.J., et al. The Atlas of European Mammals. San Diego: Academic Press. 1999.
222. Pavilinić, Igor, and Maja Đaković. The Greater Horseshoe Bat, Rhinolophus Ferrumequinum in Croatia: Present Status and Research Recommendations. Nat. Croat. Vol. 19(2), 2010. Pp 339-356.
235. Andriafidison, D, et al. 2008. Hipposideros commersoni. In: IUCN 2011. IUCN Red List of Threatened Species. Version 2011.1. <www.iucnredlist.org>. Downloaded on 08 July 2011.
239. Molur, S., Yapa, W. & Srinivasulu, C. 2008. Hipposideros lankadiva. In: IUCN 2011. IUCN Red List of Threatened Species. Version 2011.1. <www.iucnredlist.org>. Downloaded on 11 July 2011.
243. Flannery, Tim. Mammals of New Guinea. Ithaca: Cornell UP. 1995.
250. Hutterer, Rainer, Teodora Ivanova, Christine Meyer-Cords, and Luisa Rodrigues. Bat Migrations in Europe: A Review of Banding Data and Literature. Bonn: Bundesamt für Naturschutz. 2005.
251. Gopalkrishna, A. Migratory Pattern of Some Indian Bats. Myotis, 23-24. Pp. 223-227. 1985.

257. Bullen, R.D., and N.L. McKenzie. Seasonal range variation of Tadarida australis (Chiroptera: Molossidae) in Western Australia: the impact of enthalpy. Australian Journal of Zoology, 2005, 53, 145-156.
267. McKenzie, N. & Richards, G. 2008. Tadarida jobensis. In: IUCN 2011. IUCN Red List of Threatened Species. Version 2011.1. <www.iucnredlist.org>. Downloaded on 12 July 2011.
288. Dietz, Christian, Otto von Helversen, and Dietmar Nill. Bats of Britain, Europe and Northwest Africa. London: A & C Black, 2009.
314. Dávalos, L. & Tejedor, A. 2008. Natalus stramineus. In: IUCN 2011. IUCN Red List of Threatened Species. Version 2011.1. <www.iucnredlist.org>. Downloaded on 13 July 2011.
319. Adams, James K. Pteronotus davyi. Mammalian Species, No. 346. 26 October 1989.
356. Reid, Fiona A. A Field Guide to the Mammals of Central America & Southeast Mexico. New York: Oxford UP. 1997.
358. Ochoa, J. & Velazco, P. 2008. Glyphonycteris daviesi. In: IUCN 2011. IUCN Red List of Threatened Species. Version 2011.1. <www.iucnredlist.org>. Downloaded on 18 July 2011.
361. Soriano, P. & Molinari, J. 2008. Leptonycteris curasoae. In: IUCN 2011. IUCN Red List of Threatened Species. Version 2011.1. <www.iucnredlist.org>. Downloaded on 18 July 2011.
362. Cole, F. Russell, and Don E. Wilson. Leptonycteris curasoae. Mammalian Species, No. 796. 9 August 2006.
363. Hensley, Adele Pfrimmer, and Kenneth T. Wilkins. Leptonycteris nivalis. Mammalian Species, No. 307. 30 June 1988.
364. Cole, F. Russell, and Don E. Wilson. Leptonycteris yerbabuenae. Mammalian Species, No. 797. 9 August 2006.
387. Tellez, Guillermo, Rodrigo A. Medellin, Claudia Mora, and Gary McCracken. Evidence of Migration of Leptonycteris curasoae in the Mexican Tropics. Bat Research News, Vol. 41, Iss. 4. Pp 143. Sep 2000.
404. Mello, Marco A. R., Elisabeth K. V. Kalko, and Wesley R. Silva. Diet and Abundance of the Bat Surnira Lilium (Chiroptera) in a Brazilian Montane Atlantic Forest. Journal of Mammalogy, 89(2): 485-492, 2008.
418. Butchkoski, Calvin M., and John D. Chenger. Spring Indiana Bat Myotis sodalis Migration Telemetry in Pennsylvania. Bat research news. Vol. 49. Iss 4. Pp 107. 2008.
420. Bates, Paul J. J. Bats of the Indian Subcontinent. Sevenoaks, Kent, England: Harrison Zoological Museum, 1997.
434. Gonzalez, E., Barquez, R. & Miller, B. 2008. Lasiurus blossevillii. In: IUCN 2011. IUCN Red List of Threatened Species. Version 2011.1. <www.iucnredlist.org>. Downloaded on 29 July 2011.
435. Arroyo-Cabrales, J., Miller, B., Reid, F., Cuarón, A.D. & de Grammont, P.C. 2008. Lasiurus borealis. In: IUCN 2011. IUCN Red List of Threatened Species. Version 2011.1. <www.iucnredlist.org>. Downloaded on 29 July 2011.
454. Appleton, B. 2008. Miniopterus oceanensis. In: IUCN 2011. IUCN Red List of Threatened Species. Version 2011.1. <www.iucnredlist.org>. Downloaded on 01 August 2011.
455. Chiozza, F. 2008. Miniopterus fuliginosus. In: IUCN 2011. IUCN Red List of Threatened Species. Version 2011.1. <www.iucnredlist.org>. Downloaded on 01 August 2011.
475. Arroyo-Cabrales, J. & Ticul Alvarez Castaneda, S. 2008. Myotis lucifugus. In: IUCN 2011. IUCN Red List of Threatened Species. Version 2011.1. <www.iucnredlist.org>. Downloaded on 01 August 2011.
509. Pētersons, Gunārs. Seasonal migrationg of north-eastern populations of Nathusius' bat Pipistrellus nathusii (Chiroptera). Myotis. Vol 41-42. Pp 29-56. 2003.
510. Winhold, Lisa. Aspects of migration by the endangered Indiana bat, Myotis sodalis. Bat research news. 47(1):1-6. 2006.
511. Larsen, Thor. Capturing, Handling, and Marking Polar Bears in Svalbard. The Journal of Wildlife Management, Vol. 35, No. 1 (Jan., 1971), pp. 27-36.
512. Paetkau, D., et al. Genetic structure of the world's polar bear populations. Molecular Ecology(1999) 8, 1571-1584.
529. Pierce, Becky M., et al. Migratory Patterns of Mountain Lions: Implications for Social Regulation and Conservation. Journal of Mammalogy, Vol. 80, No. 3 (Aug., 1999), pp. 986-992.
533. Heide-Jorgensen, Mads Peter, and Randall R. Reeves. Evidence of a decline in beluga, Delphinapterus leucas, abundance off West Greenland. ICES J of Marine Science, 53: 61-72. 1996.
537. Mech, L.D. & Boitani, L. 2010. Canis lupus. In: IUCN 2011. IUCN Red List of Threatened Species. Version 2011.2. <www.iucnredlist.org>. Downloaded on 01 February 2012.
538. Berger, Joel. The Last Mile: How to Sustain Long-Distance Migration in Mammals. Conservation Biology, Vol 18, No. 2. Pp. 320-331. April 2004.
539. Kahurananga, James, and Frank Silkiluwasha. The migration of zebra and wildebeest betweeen Tarangire National Park and Simanjiro Plains, northern Tanzania, in 1972 and recent trends. Afr. J. Ecol. 1997, Volume

35, pages 179–185.
542. Churcher, C. S. Equus grevyi. Mammalian Species. No. 453. 15 November, 1993.
543. Estes, Richard Despard. The Behavior Guide to African Mammals. Berkeley: U California P. 1991.
668. Adrian Forsyth Mammals of North America: Temperate and Arctic Regions. Buffalo, NY: Firefly Books. 1999.
778. M. C. Crawley. Movements and Home-Ranges of Clethrionomys glareolus Schreber and Apodemus sylvaticus L. in North-East England. Oikos, Vol. 20, No. 2 (1969), pp. 310-319
779. Bo Bergstedt. Home Ranges and Movements of the Rodent Species Clethrionomys glareolus (Schreber), Apodemus flavicollis (Melchior) and Apodemus sylvaticus (Linné) in Southern Sweden. Oikos, Vol. 17, No. 2 (1966), pp. 150-157
1005. C. N. Bronner and J. A. J. Meester. Otomys angoniensis. MAMMALIAN SPECIES No. 306, pp. 1-6, 5 figs.
1027. Douglas C. Andersen, Kenneth R. Wilson, Michael S. Miller, and Miles Falck. MOVEMENT PATTERNS OF RIPARIAN SMALL MAMMALS DURING PREDICTABLE FLOODPLAIN INUNDATION. Journal of Mammalogy, 81(4):1087-1099. 2000.
1114. Victor H. Cahalane. Mammals of the Chiricahua Mountains, Cochise County, Arizona. Journal of Mammalogy, Vol. 20, No. 4 (Nov., 1939), pp. 418-440
1200. Bekenov et al. The ecology and management of the Saiga antelope in Kazakhstan. Mammal Rev (1998) vol. 28 (1) pp. 1-52
1201. Leimgruber et al. Spatial patterns in relative primary productivity and gazelle migration in the eastern steppes of Mongolia. Biol Conserv (2001) vol. 102 (2) pp. 205-212
1202. Danilkin A (1996) Behavioral ecology of Siberian and European roe deer. Wildlife Ecology and Behavior Series 2. Chapman & Hall, London
1203. Boone et al. Serengeti wildebeest migratory patterns modeled from rainfall and new vegetation growth. Ecology (2006) vol. 87 (8) pp. 1987-1994
1205. Wolfgang von Richter (1974) Connochaetes gnou. MAMMALIAN SPECIES No. 50, pp. 1-6.
1206. Paul R. Krausman and Anne L. Casey (2007) Addax nasomaculatus. MAMMALIAN SPECIES No. 807, pp. 1–4.
1207. Albert W. Franzmann (1981) Alces alces. MAMMALIAN SPECIES No. 154, pp. 1-7.
1208. Bart W. O'Gara (1978) Antilocapra americana. MAMMALIAN SPECIES No. 90, pp. 1-7.
1209. Mary Meagher (1986). Bison bison. MAMMALIAN SPECIES No. 266, pp. 1-8
1210. Alexander K. Fedosenko and David A. Blank (2001) Capra sibirica. MAMMALIAN SPECIES No. 675, pp. 1–13.
1211. Yoram Yom-Tov, Heinrich Mendelssohn, and Colin P. Groves (1995) Gazella dorcas. MAMMALIAN SPECIES No. 491, pp. 1-6.
1212. Steven C. Kingswood and David A. Blank (1996) Gazella subgutturosa. MAMMALIAN SPECIES No. 518, pp. 1-10.
1213. Allen E. Anderson and Olof C. Wallmo (1984) Odocoileus hemionus. MAMMALIAN SPECIES No. 219, pp. 1-9, 3 figs.
1214. Winston Paul Smith (1991) Odocoileus virginianus. MAMMALIAN SPECIES No. 388, pp. 1-13.
1215. Chester B. Rideout and Robert S. Hoffmann (1975) Oreamnos amencanus. MAMMALIAN SPECIES No. 63.
1216. Alexander K. Fedosenko and David A. Blank (2005) Ovis ammon. MAMMALIAN SPECIES No. 773.
1217. David M. Shackleton (1985) Ovis canadensis. MAMMALIAN SPECIES No. 230, pp. 1-9.
1218. R. Terry Bowyer and David M. Leslie, Jr. (1992) Ovis daHi. MAMMALIAN SPECIES No. 393, pp. 1-7.
1219. DAVID M. LESLIE, JR. AND GEORGE B. SCHALLER (2008 Pantholops hodgsonii (Artiodactyla: Bovidae) MAMMALIAN SPECIES 817:1–13
1220. Bergerud et al. Spring migration and dispersion of Woodland Caribou at calving. Anim Behav (1990) vol. 39 pp. 360-368
1221. Gary G. Gray and C. David Simpson (1980) Ammotragus lervia. MAMMALIAN SPECIES No. 144, pp. 1-7.
1222. Mallon, D.P. & Cuzin, F. 2008. Gazella cuvieri. In: IUCN 2011. IUCN Red List of Threatened Species. Version 2011.2. <www.iucnredlist.org>. Downloaded on 27 April 2012.
1223. David M. Forsyth. Long-term harvesting and male migration in a New Zealand population of Himalayan tahr Hemitragus jemlahicus. Journal of Applied Ecology. Volume 36, Issue 3, pages 351–362, June 1999.
1224. Jiménez, J., Guineo, G., Corti, P, Smith, J.A., Flueck, W., Vila, A., Gizejewski, Z., Gill, R., McShea, B. & Geist, V. 2008. Hippocamelus bisulcus. In: IUCN 2011. IUCN Red List of Threatened Species. Version 2011.2. <www.iucnredlist.org>. Downloaded on 27 April 2012.
1226. IUCN SSC Antelope Specialist Group 2008. Nanger granti. In: IUCN 2011. IUCN Red List of Threatened Species. Version 2011.2. <www.iucnredlist.org>. Downloaded on 27 April 2012.

1228. Timmins, R.J. & Duckworth, J.W. 2008. Rucervus eldii. In: IUCN 2011. IUCN Red List of Threatened Species. Version 2011.2. <www.iucnredlist.org>. Downloaded on 27 April 2012.
1230. Helmut Hemmer (1972) Uncia uncia. MAMMALIAN SPECIES No. 20, pp. 1-5.
1231. Garshelis, D.L. & Steinmetz, R. 2008. Ursus thibetanus. In: IUCN 2011. IUCN Red List of Threatened Species. Version 2011.2. <www.iucnredlist.org>. Downloaded on 28 April 2012.
1232. Vedder, A. L. Movement Patterns of a Group of Free-ranging Mountain Gorillas (Gorilla gorilla beringei) and Their Relation to Food Availability. American Journal of Primatology 75'3-88 (1984)
1233. Kawanishi, K., Gumal, M. & Oliver, W. 2008. Sus barbatus. In: IUCN 2011. IUCN Red List of Threatened Species. Version 2011.2. <www.iucnredlist.org>. Downloaded on 28 April 2012.
1235. Harris, R.B. & Leslie, D. 2008. Bos mutus. In: IUCN 2011. IUCN Red List of Threatened Species. Version 2011.2. <www.iucnredlist.org>. Downloaded on 28 April 2012.
1251. R.C. Trout. A review of studies on populations of wild Harvest mice (Micromys minutus (Pallas»). Mammal Review 8(4): 143-158, 1978.
1253. Madsen, Thomas and Richard Shine. Seasonal migration of predators and prey - A study of pythons and rats in tropical Australia. Ecology 77(1): 149-156, 1996.
1254. Khamphoukeo K, Bounneuang Douangboupha, Aplin KA, Singleton GR. 2003. Pest and non-pest rodents in the upland agricultural landscape of Laos: a progress report. In: Singleton GR, Hinds LA, Krebs CJ, Spratt DM, eds. Rats, mice and people: rodent biology and management. Canberra (Australia): Australian Centre for International Agricultural Research. p 284-289.
1408. Tsytsulina, K., Formozov, N. & Sheftel, B. 2008. Lemmus sibiricus. In: IUCN 2012. IUCN Red List of Threatened Species. Version 2012.1. <www.iucnredlist.org>. Downloaded on 19 August 2012.
1429. Linzey, A.V. & NatureServe (Hammerson, G.) 2008. Microtus montanus. In: IUCN 2012. IUCN Red List of Threatened Species. Version 2012.1. <www.iucnredlist.org>. Downloaded on 20 August 2012.
1446. Zhibin ZHANG, Lei XU, Cong GUO, Yong WANG and Yongwang GUO. Effect of ENSO-driven precipitation on population irruptions of the Yangtze vole Microtus fortis calamorum in the Dongting Lake region of China. Integrative Zoology 2010; 5: 176-184.
1741. Troy L. Best and Suzanne Riedel. 1995. Sciurus arizonensis. Mammalian Species 496: 1-5.
1748. John L. Koprowski. 1994. Sciurus niger. Mammalian Species 479: 1-9.
1949. Karen McBee and Robert J. Baker. 1982. Dasypus novemcinctus. Mammalian Species 162, pp. 1-9.
2048. Linda S. Broome. Density, home range, seasonal movements and habitat use of the mountain pygmy-possum Burramys parvus (Marsupialia: Burramyidae) at Mount Blue Cow, Kosciuszko National Park. Austral Ecology (2001) 26, 275–292.
2051. Jamie M. Harris. 2009. Cercartetus lepidus (Diprotodontia: Burramyidae). Mammalian Species 842:1-11.
2185. Larry Laursen and Marc Bekoff. 1978. Loxodonta africana. Mammalian Species 92:1-8.
2186. P. J. Viljoen. Spatial distribution and movements of elephants (Loxodonta africana) in the northern Namib Desert region of the Kaokoveld, South West Africa/Namibia. J. Zool Lond. (1989) 219, 1-19.
2249. R. Hewson and M. D. C. Hinge. 1990. Characteristics of the Home Range of Mountain Hares Lepus timidus. Journal of Applied Ecology 27: 651-666.
2254. By Troy L. Best. 1996. Lepus californicus. Mammalian Species 530: 1-10.
2846. Leslie N. Carraway, Lois F. Alexander, and B. J. Verts. 1993. Scapanus townsendii. Mammalian Species 434: 1-7.
2878. Michael J. Casimir and Eckart Butenandi. 1973. Migration and Core Area Shifting in Relation to some Ecological Factors in a Mountain Gorilla Group (Gorilla gorilla beringeir) in the Mt. Kahuzi Region (Republique du Zaire)). Zeitschrift für Tierpsychologie 33: 514-522.
2929. Friederike Scholz and Peter M. Kappeler. 2004. Effects of SeasonalWater Scarcity on the Ranging Behavior of Eulemur fulvus rufus. International Journal of Primatology 25(3): 599-613.
2933. Lennart W. Pyritz, Peter M. Kappeler, Claudia Fichtel. 2011. Coordination of Group Movements in Wild Red-fronted Lemurs (Eulemur rufifrons): Processes and Influence of Ecological and Reproductive Seasonality. Int J Primatol 32: 1325–1347.
3046. de la Torre, S., Morales, A. L., Link, A. & Cornejo, F. 2008. Cebus albifrons. In: IUCN 2012. IUCN Red List of Threatened Species. Version 2012.2. <www.iucnredlist.org>. Downloaded on 27 May 2013.
3074. Peres, C. A. (1994). Primate responses to phenological changes in an Amazonian terra firme forest. Biotropica 26: 98–112.
3075. Adrian A. Barnett, Carol Volkmar de Castilho, Rebecca L. Shapley, and Antenor Anicacio. 2005. Diet, Habitat Selection and Natural History of Cacajao melanocephalus ouakary in Jau National Park, Brazil. International Journal of Primatology 26(4): 949-969.

3076. Eckhard W. Heymann, Rolando Aquino. 2010. Peruvian Red Uakaris (Cacajao calvus ucayalii) Are Not Flooded-Forest Specialists. International Journal of Primatology 31(5): 751-758.
3162. Hiroyuki Takasaki. 1981. Troop Size, Habitat Quality, and Home Range Area in Japanese Macaques. Behav Ecol Sociobiol 9: 277-281.
3208. Li, B. G., Chen, C., Ji, W. H., & Ren, B. P. (1999). Seasonal home range changes of the Sichuan snubnosed monkey (Rhinopithecus roxellana) in the Qinling Mountains of China. Folia Primatologica 71 375–386.
3209. Wada, K., & Ichiki, Y. (1980). Seasonal home range use by Japanese monkeys in the Shiga Heights. Primates 21 468–483.
3210. Rui-Chang Quan, Guopeng Ren, Jocelyn E. Behm3 Lin Wang, Yong Huang, Yongcheng Long, Jianguo Zhu. 2011. Why Does Rhinopithecus bieti Prefer the Highest Elevation Range in Winter? A Test of the Sunshine Hypothesis. PLoS ONE 6(9) e24449.
3254. te Boekhoerst, I. J. A., Schurmann, V. L., and Sugardjito, J. (1990). Residential status and seasonal movement of wild orangutans in the Gunung Leuser Reserve (Sumatra, Indonesia). Anim. Behav. 39: 1098-1109.
3259. JM Fryxell, ARE Sinclair. 1988. Causes and consequences of migration by large herbivores. TREE 3(9):237-241.
3260. Geist, V., Ferguson, M. & Rachlow, J. 2008. Alces americanus. In: IUCN 2012. IUCN Red List of Threatened Species. Version 2012.2. <www.iucnredlist.org>. Downloaded on 12 June 2013.
3267. Timmins, R., Duckworth , J.W., Samba Kumar, N., Anwarul Islam, M., Sagar Baral, H., Long, B. & Maxwell, A. 2012. Axis porcinus. In: IUCN 2012. IUCN Red List of Threatened Species. Version 2012.2. <www.iucnredlist.org>. Downloaded on 12 June 2013.
3272. Mysterud, Atle. 1999. Seasonal migration pattern and home range of roe deer (Capreolus capreolus) in an altitudinal gradient in southern Norway. J. Zool., Lond. 247:479-486.
3273. Herrero, J., I. Garb, A. Garcia-Serrano, R. Garcia-Gonzilez. 1996. Habitat use in a Rupicapra pyrenaica pyrenuica forest population. Forest Ecology and Management 88:25-29.
3274. Aulagnier, S., Giannatos, G. & Herrero, J. 2008. Rupicapra rupicapra. In: IUCN 2012. IUCN Red List of Threatened Species. Version 2012.2. <www.iucnredlist.org>. Downloaded on 12 June 2013.
3297. Walfrido M. Tomas & Suzana M. Salis (2000): Diet of the marsh deer (Blastocerus dichotomus) in the Pantanal wetland, Brazil, Studies on Neotropical Fauna and Environment, 35:3, 165-172
3304. Tian-Pei Guan, Bao-Ming Ge, William J. McShea, Sheng Li, Yan-Ling Song, Chad M. Stewart. 2013. Seasonal migration by a large forest ungulate: a study on takin (Budorcas taxicolor) in Sichuan Province, China. European Journal of Wildlife Research 59(1): 81-91.
3305. Hare, J. 2008. Camelus ferus. In: IUCN 2012. IUCN Red List of Threatened Species. Version 2012.2. <www.iucnredlist.org>. Downloaded on 14 June 2013.
3307. Weinberg, Paul J. 2002. Capra cylindricornis. Mammalian Species 695: 1-9.
3312. Igota, H., Sakuragi, M., Uno, H., Kaji, K., Kaneko, M., Adamatsu, R. and Maekawa, K. (2004), Seasonal migration patterns of female sika deer in eastern Hokkaido, Japan. Ecological Research, 19: 169–178.
3314. Nelson, Michael E. and L. David Mech (1984). Home-Range Formation and Dispersal of Deer in Northeastern Minnesota. Journal of Mammalogy 65(4): 567-575.
3321. D. Blank , K.E. Ruckstuhl & W. Yang (2012): Social organization in goitered gazelle (Gazella subgutturosa Güldenstaedt 1780), Ethology Ecology & Evolution, 24:4, 306-321
3327. Fischer, F. and Linsenmair, K. E. (2001), Spatial and temporal habitat use of kob antelopes (Kobus kob kob, Erxleben 1777) in the Comoé National Park, Ivory Coast as revealed by radio tracking. African Journal of Ecology, 39: 249–256. doi: 10.1046/j.0141-6707.2000.00302.x
3328. Fryxell, J. M., and A. R. E. Sinclair. 1988. Seasonal migration by white-eared kob in relation to resources. African Journal Of Ecology 26:17–31.
3330. Puiga, Silvia, María I. Rosi, Fernando Videla, Eduardo Mendez. 2011. Summer and winter diet of the guanaco and food availability for a High Andean migratory population (Mendoza, Argentina). Mammalian Biology 76 727–734.
3370. Duckworth, J.W. & MacKinnon, J. 2008. Naemorhedus goral. In: IUCN 2012. IUCN Red List of Threatened Species. Version 2012.2. <www.iucnredlist.org>. Downloaded on 18 June 2013.
3380. Gunn, A. & Forchhammer, M. 2008. Ovibos moschatus. In: IUCN 2012. IUCN Red List of Threatened Species. Version 2012.2. <www.iucnredlist.org>. Downloaded on 18 June 2013.
3390. Leslie Jr., David M. 2009. Przewalskium albirostre. Mammalian Species 42(849):7-18.
3391. Wilson, P., 1^81. - Ecology and habitat utilization of blue sheep Pseudois navaur in Nepal. Biol. Consent, 21 : 55-74.
3392. Oli, M. K. 1996. Seasonal patterns in habitat use of blue sheep Pseudois nayaur (Artiodactyla, Bovidae) in

Nepal. Mammalia 60(2): 187-193.
3461. V Utreras B, ER Suárez R, GR Zapata-Ríos, G Lasso, L Pinos. 2005. Dry and rainy season estimations of giant otter, Pteronura brasiliensis, home range in the Yasuní National Park, Ecuador. LAJAM 4(2): 191-194.
3463. Durant, S. M., Caro, T. M., Collins, D. A., Alawi, R. M. and Fitzgibbon, C. D. 1988. Migration patterns of Thomson's gazelles and cheetahs on the Serengeti Plains. African Journal of Ecology 26: 257.
3464. Durant, S., Marker, L., Purchase, N., Belbachir, F., Hunter, L., Packer, C., Breitenmoser-Wursten, C., Sogbohossou, E. & Bauer, H. 2008. Acinonyx jubatus. In: IUCN 2012. IUCN Red List of Threatened Species. Version 2012.2. <www.iucnredlist.org>. Downloaded on 26 June 2013.
3485. Jackson, R., Mallon, D., McCarthy, T., Chundaway, R.A. & Habib, B. 2008. Panthera uncia. In: IUCN 2012. IUCN Red List of Threatened Species. Version 2012.2. <www.iucnredlist.org>. Downloaded on 27 June 2013.
3497. Courtenay, O. & Maffei, L. 2008. Cerdocyon thous. In: IUCN 2012. IUCN Red List of Threatened Species. Version 2012.2. <www.iucnredlist.org>. Downloaded on 01 July 2013.
3640. Angerbjörn, Anders and John E. C. Flux (1995) Lepus timidus. Mammalian Species 495: 1-11.
3641. Smith GW, Stoddart LC, Knowlton FF (2002) Long-distance movements of black-tailed jackrabbits. Journal of Wildlife Management 66(2): 463–469.
3642. Walton LR, Cluff HD, Paquet PC, Ramsay MA (2001) Movement patterns of barren-ground wolves in the central canadian arctic. Journal of Mammalogy 82(3): 867–876
3643. Mead, J. I. (1989) Nemorhaedus goral. Mammalian Species 335: 1-5
3644. Jones, J. K. and C. S. Hood. 1993. Synopsis of South American Bats of the Family Emballonuridae. Occasional Papers The Museum Texas Tech University, 155: 1-32. Accessed 15 July 2013 at http://www.loyno.edu/~chood/joneshood1993.pdf.
3645. Nordoy, B., L. Folkow, A. Blix. 1995. Distribution and diving behaviour of crabeater seals (Lobodon carcinophagus) off Queen Maud Land. Polar Biology, 15: 261-268. http://link.springer.com/article/10.1007/BF00239846
3646. Baker R (1978) The evolutionary ecology of animal migration (Holmes and Meier, New York).
3647. Groves, C. P. 1972 Ceratotherium simum. Mammalian Species 8: 1-6.
3648. Cotterill, F., R. Fergusson. 1999. Reproductive ecology of Commerson's leaf-nosed bats Hipposideros commersoni (Chiroptera: Hipposideridae) in South-Cental Africa: interactions between seasonality and large body sizes; and implications for conservation. South African Journal of Zoology, 34/1: 53-63.
3649. Culik, Boris (2010) Odontocetes. The toothed whales: “Sotalia fluviatilis”. UNEP/CMS Secretariat, Bonn, Germany. http://www.cms.int/reports/small_cetaceans/data/S_fluviatilis/s_fluviatilis.htm (downloaded 25 July 2013)
3651. L. Karczmarski , V. G. Cockcroft & A. McLachlan (1999) Group size and seasonal pattern of occurrence of humpback dolphins Sousa chinensis in Algoa Bay, South Africa, South African Journal of Marine Science, 21:1, 89-97, DOI: 10.2989/025776199784126024
3653. Banikov A (1976) Wild camels of the gobi. Wildlife 18:398–403
3654. Roberts, T. J. 1977. The Mammals of Pakistan. Pakistan, London : Ernest Benn.
3655. Whitaker, J., W. Hamilton. 1998. Mammals of the Eastern United States. Ithaca, New York: Cornell University Press.
3657. M. Carolina Caceres and Robert M. R. Barclay. Mammalian Species, No. 634, Myotis septentrionalis (May 12, 2000), pp. 1-4
3658. Kevin E. McHugh, Robert C. Mings (2010). On the road again: Seasonal migration to a sunbelt metropolis. Urban Geography 1-18

Table S2. *D* (Fritz & Purvis 2010) values for migration and each of the migration types (breeding, refuge, tracking) across only migrating species. The mean and standard deviation values of *D* across all bootstrap phylogenies are given. The last four rows indicate the percentage of times that a significant *p*-value was detected (at the level indicated) in all phylogenies. E.g. 100% for P(*D*=0)<=0.01 means in all bootstraps, the *D* values for those migration types were significantly not zero at α=0.01 (two-tailed; bootstrapped). The interpretation of *D* values is described in the Methods section.

	**Migration (overall)**	**Breeding (among migrants)**	**Refuge (among migrants)**	**Tracking (among migrants)**
***D* - mean**	0.42	0.02	0.40	0.46
***D* - SD**	0.01	0.04	0.02	0.02
**# models successful**	666	666	666	666
**% P(*D*=0)<=0.05**	100.00	0.00	99.70	100.00
**% P(*D*=0)<=0.01**	100.00	0.00	7.96	62.93
**% P(*D*=1)<=0.05**	100.00	100.00	100.00	100.00
**% P(*D*=1)<=0.01**	100.00	100.00	100.00	100.00

Table S3. Number of migratory species by each locomotion and migration type, see also Figure S1.

	Walking	Swimming	Flying	Total
**Breeding**	1	24	5	30
**Refuge**	51	16	41	108
**Tracking**	40	16	11	67
**Total**	92	56	57	205

Table S4. The fraction of shared branch length (Shared) and the corresponding standard effect size (SES) for each migration type by locomotion type. The contents are described in the Methods section.

	**Walking**		**Swimming**		**Flying**	
	**Shared**	**SES**	**Shared**	**SES**	**Shared**	**SES**
**Breeding**	0.045	-3.560	0.483	3.118	0.290	0.399
**Refuge**	0.248	0.576	0.275	-2.098	0.397	1.338
**Tracking**	0.201	0.802	0.323	-0.290	0.231	-2.953

Table S5. Phylogenetic logistic regressions (Ho & Ané 2014) of migration across all mammals for which we had sufficient data (158 migrants, 965 mammal species in total). All categorical variable estimates, bar the intercept, are statistical contrasts: they are the difference in overall mean for that group from the overall mean of a species with one habitat-type, that is herbivorous, that has one dietary category, and walks. The regression was run two ways: once with IUCN Red List as numeric, and once as binary (threatened vs not). Estimates are reported as means and standard deviations across the 984 bootstrap phylogenies for the first regression and 989 for the second; models in 16 (and 11, respectively) phylogenies had numerical fitting problems (as reported by `phylolm`; Ho & Ané 2014) and their estimates are not reported. The mean and standard deviation of the α parameter, which reports the estimated error due to phylogenetic co-variation, was 0.0124 ± 0.00263 for the first regression and 0.01± 0.0027 for the second. Asterisks (*) indicate factors significant at the 0.05 level; for the presentation of coefficients and their associated standard deviations we follow the advice of Cole (2015), while for *p*-values we present at three decimal places following the defaults in the `R` (R Core Team 2014) statistical software. Coefficients with large standard errors and/or non-significant average p-values suggest explanatory variables for which there is little evidence of an association with migration.

	Red List category as numeric			Red List category as binary factor		
	Estimate (mean ± SD)	Std Err (mean ± SD)	*p*-value (mean ± SD)	Estimate (mean ± SD)	Std Err (mean ± SD)	*p*-value (mean ± SD)
Intercept *(1 habitat, herbivore, 1 dietary category,*	-5.98 ± 0.9265	1.07 ± 0.0961	0 ± 0			

*walking) **						
Intercept<br>*(1 habitat, herbivore, 1 dietary category, walking, not Red Listed as threatened) **				-5.89 ± 0.9272	1.06 ± 0.0966	<0.0001 ± 0.0002
log10(body mass) *	1.19 ± 0.1559	0.18 ± 0.0191	0 ± 0	1.16 ± 0.157	0.17 ± 0.0191	<0.0001 ± <0.0001
2 habitat	0.14 ± 0.1137	0.25 ± 0.0226	0.528 ± 0.1702	0.12 ± 0.1078	0.25 ± 0.0217	0.5717 ± 0.1758
3 habitats	-14.46 ± 0.3017	741.01 ± 152.1249	0.983 ± 0.007	-14.5 ± 0.2942	719.68 ± 148.2678	0.9826 ± 0.0066
4 habitats	-13.89 ± 0.3017	3546.95 ± 797.7353	0.997 ± 0.0017	-13.83 ± 0.2942	3375.24 ± 766.3315	0.9964 ± 0.0016
Omnivore	0.6 ± 0.0965	0.35 ± 0.0338	0.098 ± 0.0502	0.61 ± 0.0963	0.34 ± 0.0335	0.0864 ± 0.0469
Carnivore	0.41 ± 0.2105	0.4 ± 0.0254	0.313 ± 0.1943	0.36 ± 0.1927	0.4 ± 0.0248	0.3586 ± 0.1847
2 dietary categories	0.51 ± 0.1048	0.3 ± 0.0193	0.113 ± 0.1019	0.46 ± 0.1105	0.3 ± 0.0192	0.1494 ± 0.1258
3 dietary categories	0.32 ± 0.1121	0.39 ± 0.03	0.427 ± 0.1621	0.27 ± 0.1081	0.38 ± 0.0316	0.4955 ± 0.1626
4 dietary categories	0.21 ± 0.1019	0.41 ± 0.031	0.606 ± 0.1331	0.19 ± 0.1015	0.41 ± 0.0331	0.6204 ± 0.129

5 dietary categories	0.6 ± 0.1591	0.48 ± 0.0403	0.232 ± 0.1213	0.53 ± 0.1655	0.47 ± 0.0423	0.2869 ± 0.1489
6 dietary categories	-0.4 ± 0.1285	0.51 ± 0.04	0.444 ± 0.1443	-0.42 ± 0.1361	0.5 ± 0.0407	0.4154 ± 0.1526
7 dietary categories	0.65 ± 0.1136	0.89 ± 0.0717	0.466 ± 0.0801	0.65 ± 0.1307	0.88 ± 0.0731	0.462 ± 0.0961
8 dietary categories	-13 ± 0.3017	1749.22 ± 443.0844	0.993 ± 0.0042	-12.96 ± 0.2942	1657.13 ± 424.7377	0.9928 ± 0.0041
Red List (slope)	-0.04 ± 0.0221	0.06 ± 0.0051	0.551 ± 0.1905			
Red List (Threatened)				0.06 ± 0.0712	0.19 ± 0.0167	0.7103 ± 0.2119
Flying *	3.29 ± 0.5125	0.79 ± 0.0282	0.002 ± 0.0079	3.23 ± 0.5136	0.79 ± 0.0278	0.0015 ± 0.0059
Swimming	-0.25 ± 0.2175	0.53 ± 0.0682	0.556 ± 0.1505	-0.28 ± 0.202	0.52 ± 0.0639	0.532 ± 0.1395

Table S6. Phylogenetic logistic regressions (Ho & Ané 2014) of whether a species was a breeding migrant (n = 18) compared with all mammals we were certain were migrants of some kind (n = 113). The regression was run two ways: once with IUCN Red List as numeric, and once as binary (threatened vs not). Estimates are reported as means and standard deviations across the 939 bootstrap phylogeniesfor the first regression and 999 for the second; models in 61 (and 1, respectively) phylogenies had numerical fitting problems (as reported by `phylolm`; Ho & Ané 2014) and their estimates are not reported. The mean and standard deviation of the α parameter, which reports the estimated error due to phylogenetic co-variation, was 0.0026 ± 0.0035 for the first regression and 0.0052 ± 0.000413 for the second. See Table S4 for presentation details.

	Red List category as numeric			Red List category as binary factor		
	Estimate (mean ± SD)	Std Err (mean ± SD)	*p*-value (mean ± SD)	Estimate (mean ± SD)	Std Err (mean ± SD)	*p*-value (mean ± SD)
Intercept *(1 habitat, herbivore, 1 dietary category, walking)*	-42.37 ± 2.7545	2221.77 ± 1839.4338	0.961 ± 0.0457			
Intercept *(1 habitat, herbivore, 1 dietary category, walking, not Red Listed as Critical Endangered)*				-2.01 ± 0.1407	2 ± 0.132	0.3155 ± 0.0167

log10(body mass) *	1.77 ± 0.4244	0.82 ± 0.1589	0.035 ± 0.0166	0.00 ± 0.0205	0.22 ± 0.0152	0.9858 ± 0.0772
2 habitats	3.37 ± 1.4069	1.99 ± 0.4239	0.135 ± 0.0947	0.02 ± 0.0673	0.43 ± 0.0518	0.9702 ± 0.1227
Omnivore	19.24 ± 1.4055	2004.44 ± 1556.0773	0.980 ± 0.0314	0.02 ± 0.0686	0.43 ± 0.098	0.9787 ± 0.0889
Carnivore	16.66 ± 1.411	2092.79 ± 1761.708	0.984 ± 0.0191	0.01 ± 0.0526	0.63 ± 0.0668	0.9829 ± 0.0684
2 dietary categories	1.68 ± 0.9591	1.14 ± 0.2897	0.206 ± 0.1611	0.02 ± 0.0696	0.26 ± 0.0379	0.9644 ± 0.1439
3 dietary categories	1.07 ± 2.0398	1825.88 ± 2060.9277	0.991 ± 0.0265	0.01 ± 0.0706	0.43 ± 0.0411	0.9731 ± 0.1123
4 dietary categories	6.81 ± 1.9039	3592.57 ± 4556.7071	0.637 ± 0.3547	0.00 ± 0.0294	0.30 ± 0.0913	0.9913 ± 0.0488
5 dietary categories	3.34 ± 1.814	3237.24 ± 4133.4357	0.533 ± 0.4265	0.00 ± 0.0141	0.70 ± 0.0565	0.9971 ± 0.0169
6 dietary categories	-1.4 ± 2.772	1826.18 ± 2060.6628	0.975 ± 0.0446	0.00 ± 0.0159	0.51 ± 0.1052	0.9968 ± 0.0198
7 dietary categories	4.38 ± 1.9008	10828.64 ± 13843.1614	0.862 ± 0.1836	0.01 ± 0.0417	1.57 ± 0.1447	0.9948 ± 0.0296
Red List (slope)	-0.12 ± 0.1736	0.14 ± 0.0559	0.515 ± 0.3521			

Red List (threatened)				0.02 ± 0.0688	0.20 ± 0.0256	0.9583 ± 0.1715
Flying	21.46 ± 2.0387	1528.44 ± 1690.6905	0.884 ± 0.1499	0.03 ± 0.1111	1.58 ± 0.1376	0.9801 ± 0.0857
Swimming	12.87 ± 4.25	1528.38 ± 1690.7476	0.941 ± 0.0856	0.00 ± 0.03	1.11 ± 0.0925	0.9929 ± 0.0322

Table S7. Phylogenetic logistic regressions (Ho & Ané 2014) of whether a species was a refuge migrant (*n* = 64) compared with all mammals we were certain were migrants of some kind (*n* = 113). The regression was run two ways: once with IUCN Red List as numeric, and once as binary (threatened vs not). Estimates are reported as means and standard deviations across the 995 bootstrap phylogenies; models in 5 phylogenies had numerical fitting problems (as reported by `phylolm`; Ho & Ané 2014) and their estimates are not reported. The mean and standard deviation of the α parameter, which reports the estimated error due to phylogenetic co-variation, was 0.018 ± 0.0078 for the first regression and 0.019 ± 0.00295 for the second. See Table S4 for presentation details.

	Red List category as numeric			Red List category as binary factor		
	Estimate (mean ± SD)	Std Err (mean ± SD)	*p*-value (mean ± SD)	Estimate (mean ± SD)	Std Err (mean ± SD)	*p*-value (mean ± SD)
Intercept *(1 habitat, herbivore, 1 dietary category, walking)*	2.76 ± 0.3456	1.55 ± 0.0771	0.082 ± 0.0521			
Intercept *(1 habitat, herbivore, 1 dietary category, walking, not Red Listed as Critical Endangered)*				0.16 ± 0.1709	1.35 ± 0.0279	0.8923 ± 0.0848
log10(body mass)	-0.38 ± 0.0909	0.26 ± 0.0102	0.163 ±	-0.19 ± 0.0434	0.24 ± 0.0058	0.4319 ±

			0.0684			0.097
2 habitats	0.68 ± 0.2042	0.74 ± 0.0598	0.377 ± 0.1321	0.38 ± 0.0914	0.63 ± 0.0225	0.5462 ± 0.1013
Omnivore	-1.23 ± 0.2415	0.94 ± 0.0294	0.204 ± 0.119	0.44 ± 0.1185	0.85 ± 0.0391	0.6069 ± 0.0865
Carnivore	-1.05 ± 0.3415	0.94 ± 0.0352	0.293 ± 0.1946	0.42 ± 0.1496	0.93 ± 0.0273	0.6508 ± 0.1124
2 dietary categories	-0.11 ± 0.2235	0.54 ± 0.0286	0.783 ± 0.2387	0.00 ± 0.1137	0.48 ± 0.0362	0.8568 ± 0.1122
3 dietary categories	-0.19 ± 0.2412	0.82 ± 0.0371	0.815 ± 0.1944	-0.11 ± 0.1166	0.82 ± 0.0393	0.8768 ± 0.0904
4 dietary categories	0.17 ± 0.126	1.05 ± 0.095	0.864 ± 0.0721	0.22 ± 0.1055	0.82 ± 0.0680	0.7912 ± 0.0876
5 dietary categories	-0.46 ± 0.1179	1.02 ± 0.0746	0.652 ± 0.0776	-0.17 ± 0.1131	1.05 ± 0.0368	0.8711 ± 0.0792
6 dietary categories	0.2 ± 0.2204	1.26 ± 0.046	0.849 ± 0.1033	-0.51 ± 0.1248	1.20 ± 0.0498	0.6745 ± 0.0741
7 dietary categories	15.99 ± 0.2657	3134.53 ± 766.8089	0.996 ± 0.0013	0.70 ± 0.2041	2.12 ± 0.1299	0.7457 ± 0.0594
Red List (slope)	-0.05 ± 0.0482	0.11 ± 0.0085	0.589 ± 0.1695			
Red List (threatened)				-0.14 ± 0.1187	0.34 ± 0.0246	0.6773 ±

						0.2178
Flying	-0.36 ± 0.3285	1.31 ± 0.0702	0.760 ± 0.1584	0.41 ± 0.1049	1.17 ± 0.0117	0.7231 ± 0.0616
Swimming	-0.12 ± 0.3324	1.02 ± 0.0445	0.766 ± 0.116	0.33 ± 0.1117	1.06 ± 0.0235	0.752 ± 0.0762

Table S8. Phylogenetic logistic regressions (Ho & Ané 2014) of whether a species was a tracking migrant (*n* = 64) compared with all mammals we were certain were migrants of some kind (*n* = 113). The regression was run two ways: once with IUCN Red List as numeric, and once as binary (threatened vs not). Estimates are reported as means and standard deviations across the 942 bootstrap phylogenies; models in 58 phylogenies had numerical fitting problems (as reported by `phylolm`; Ho & Ané 2014) and their estimates are not reported. The mean and standard deviation of the α parameter, which reports the estimated error due to phylogenetic co-variation, was 0.039 ± 0.016 for the first regression and 0.0066 ± 0.00484 for the second. See Table S4 for presentation details.

	Red List category as numeric			Red List category as binary factor		
	Estimate (mean ± SD)	Std Err (mean ± SD)	*p*-value (mean ± SD)	Estimate (mean ± SD)	Std Err (mean ± SD)	*p*-value (mean ± SD)
Intercept *(1 habitat, herbivore, 1 dietary category, walking)*	-1.25 ± 0.1369	1.59 ± 0.0224	0.431 ± 0.0462			
Intercept *(1 habitat, herbivore, 1 dietary category, walking, not Red Listed as Critical Endangered)*				-0.13 ± 0.2916	1.44 ± 0.0397	0.8569 ± 0.1004
log10(body mass)	-0.12 ± 0.0465	0.27 ± 0.0061	0.654 ±	0.10 ± 0.0748	0.23 ± 0.0129	0.6395 ±

			0.1143			0.1833
2 habitats	-1.65 ± 0.1839	0.84 ± 0.0334	0.054 ± 0.0266	-0.91 ± 0.4951	0.53 ± 0.0716	0.1925 ± 0.2159
Omnivore	1.14 ± 0.1193	1.02 ± 0.0192	0.269 ± 0.0533	-0.29 ± 0.1799	0.76 ± 0.0628	0.7051 ± 0.1652
Carnivore *	3.03 ± 0.189	1.36 ± 0.0449	0.026 ± 0.0201	0.98 ± 0.5215	0.93 ± 0.0654	0.3605 ± 0.2405
2 dietary categories	0.91 ± 0.1135	0.63 ± 0.0421	0.159 ± 0.0687	0.14 ± 0.1626	0.44 ± 0.0371	0.7175 ± 0.192
3 dietary categories	1.41 ± 0.0965	1 ± 0.0314	0.161 ± 0.0329	0.07 ± 0.2071	0.76 ± 0.0674	0.8271 ± 0.1423
4 dietary categories	0.87 ± 0.1034	0.78 ± 0.1262	0.263 ± 0.0964	-0.21 ± 0.2181	0.70 ± 0.1353	0.7059 ± 0.1265
5 dietary categories	1.4 ± 0.115	1.22 ± 0.0263	0.255 ± 0.0483	0.61 ± 0.3063	0.99 ± 0.0919	0.5618 ± 0.1754
6 dietary categories	0.29 ± 0.0644	1.54 ± 0.0269	0.851 ± 0.0288	0.28 ± 0.3255	1.11 ± 0.1206	0.7772 ± 0.1989
7 dietary categories	-14.82 ± 0.2362	1867.14 ± 269.8648	0.993 ± 0.003	-0.04 ± 0.2165	1.79 ± 0.2141	0.9432 ± 0.0607
Red List (slope) *	0.36 ± 0.0436	0.15 ± 0.0074	0.017 ± 0.0238			
Red List (threatened)				0.73 ± 0.2301	0.36 ± 0.0286	0.0793 ±

						0.0909
Flying *	-4.33 ± 0.1403	1.58 ± 0.0664	0.006 ± 0.0054	-1.57 ± 0.7962	1.23 ± 0.0609	0.2846 ± 0.233
Swimming *	-2.94 ± 0.1062	1.35 ± 0.0584	0.031 ± 0.0081	-1.19 ± 0.3294	0.95 ± 0.0591	0.2343 ± 0.1413

Table S9. The median and interquartile range (IQR) values of significant life history traits and the IUCN Red List rank for each migration type (breeding, refuge, tracking).

Factor		Breeding	Refuge	Tracking
Mass (g)	Median	137194.86	3185.74	53954.05
	IQR	184679.00	75885.83	195198.60
Habitat breadth	Median	2	1	1
	IQR	0	0	0
IUCN Red List rank	Median	2	1	2
	IQR	2	1	2
